# A pattern of cognitive resource disruptions in childhood psychopathology

**DOI:** 10.1162/netn_a_00322

**Published:** 2023-10-01

**Authors:** Andrew J. Stier, Carlos Cardenas-Iniguez, Omid Kardan, Tyler M. Moore, Francisco A. C. Meyer, Monica D. Rosenberg, Antonia N. Kaczkurkin, Benjamin B. Lahey, Marc G. Berman

**Affiliations:** Department of Psychology, University of Chicago; Department of Psychiatry, University of Pennsylvania; Department of Psychology, Vanderbilt University; The Neuroscience Institute, University of Chicago; Department of Public Health Sciences, University of Chicago

**Keywords:** Psychopathology, Working memory, Hurst exponent, fMRI, Fractals, Cognition

## Abstract

The Hurst exponent (*H*) isolated in fractal analyses of neuroimaging time series is implicated broadly in cognition. Within this literature, *H* is associated with multiple mental disorders, suggesting that *H* is transdimensionally associated with psychopathology. Here, we unify these results and demonstrate a pattern of decreased *H* with increased general psychopathology and attention-deficit/hyperactivity factor scores during a working memory task in 1,839 children. This pattern predicts current and future cognitive performance in children and some psychopathology in 703 adults. This pattern also defines psychological and functional axes associating psychopathology with an imbalance in resource allocation between fronto-parietal and sensorimotor regions, driven by reduced resource allocation to fronto-parietal regions. This suggests the hypothesis that impaired working memory function in psychopathology follows from a reduced cognitive resource pool and a reduction in resources allocated to the task at hand.

## Author Summary

We study how the complexity of brain signals is associated with psychopathology. Using a measure of the fractalness of temporal brain dynamics, we find that psychopathology is associated with less fractal and less complex brain signals globally. In addition to this global pattern, we investigate spatial variations in the fractalness of brain signals. We find that disruptions in cognitive resources during a working memory task come from reductions in resources allocated to frontal regions alone. In addition, we find that these patterns of resource allocation are predictive of cognitive performance on multiple working memory tasks and a future working memory task. These findings suggest that future investigations of fractal brain dynamics in other contexts may help researchers better understand the mechanisms behind psychopathology-associated cognitive impairment.

## INTRODUCTION

Fractals are found everywhere in the natural world. These patterns are pervasive and include the growth of Romanesco broccoli, the shape of coastlines (Mandelbrot, 1967), and even the time series of human neuroimaging data (Churchill et al., 2015; He, 2011; Kardan et al., 2023; Suckling, Wink, Bernard, Barnes, & Bullmore, 2008). Interestingly, fractal brain dynamics measured with human neuroimaging have been linked to numerous mental disorders and related traits, including depression (Čukić et al., 2020; Gao, Chen, Biswal, Lei, & Yuan, 2018; Wei et al., 2013), drug addiction (Ide, Hu, Zhang, Mujica-Parodi, & Chiang-shan, 2016), schizophrenia (Sokunbi et al., 2014), ADHD (Sokunbi, 2018), autism (Lai et al., 2010), and impulsivity (Akhrif, Romanos, Domschke, Schmitt-Boehrer, & Neufang, 2018). While these studies have converged on the finding that psychopathology is associated with ‘less fractal’ and more random, brain dynamics, they have not provided mechanistic insight into these associations or helped to clarify why psychopathology is associated with poor cognitive task performance. However, recent evidence suggests that fractal measures of brain dynamics may quantify how cognitive resources are utilized during tasks (Churchill et al., 2015; Kardan et al., 2020, 2023; Suckling et al., 2008). Building on these advances, here, we investigate whether fractal brain dynamics can advance a mechanistic understanding—at the functional phenomenology level of analysis (Bassett, Zurn, & Gold, 2018)—of the relationship between psychopathology and cognition.

**Figure 1.**
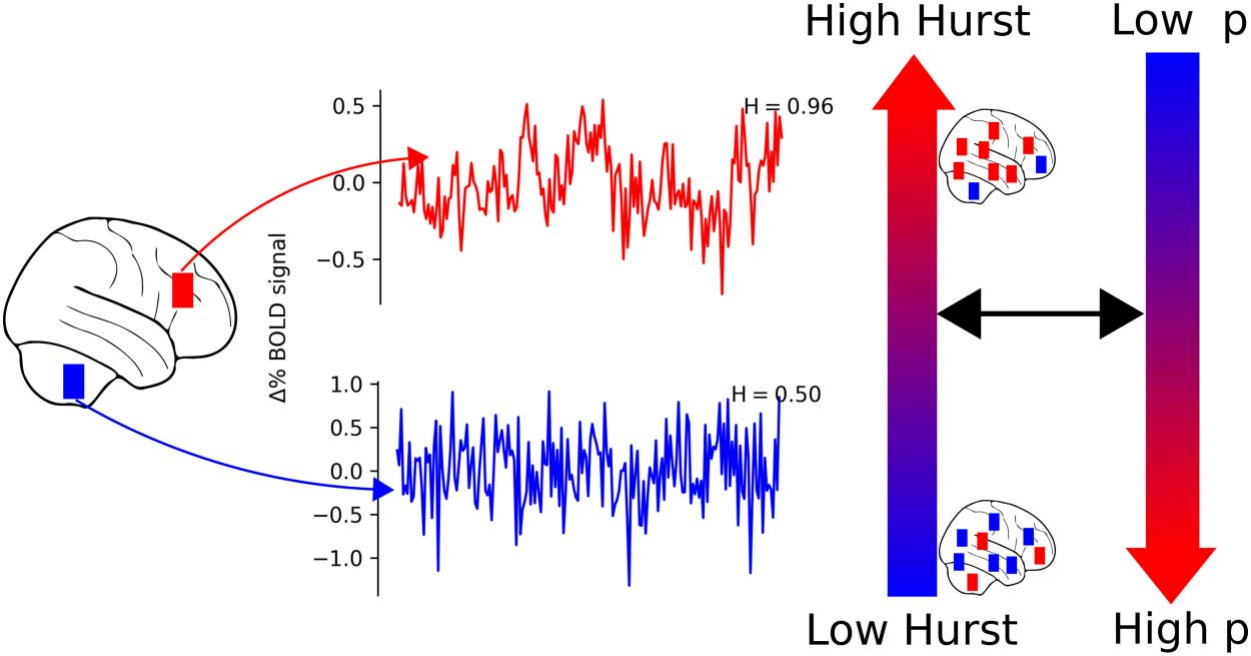
***Hypothesis 1***. Time series with *H* close to 1 have smooth-looking temporal fluctuations (top). Such time series can also be described as scale-free or fractal in time. In contrast, time series with more random temporal fluctuations have *H* closer to 0.5 (bottom). We hypothesized that higher *H* would be associated with lower extracted factor scores for a general factor of psychopathology, p. Though we characterize individuals as having lower *H* or higher *H*, there is still variability in *H* across brain regions.

The defining characteristic of fractals is scale-invariance, which refers to the fact that fractals look the same across levels of magnification: fractal shapes look the same zoomed in versus zoomed out. In other words, fractals display self-similarity. In the context of human neuroimaging data, fractalness is commonly estimated by the Hurst exponent (Peng et al., 1994), *H* (see Figure 1), and refers to the degree to which the collected time series (e.g., from blood oxygen level-dependent functional magnetic resonance imaging, BOLD fMRI, or electroencephalography) are scale-free, or self-similar, in time. Values of *H* close to 1 are observed from signals that have high fractalness and self-similarity; lower values of *H*, close to 0.5, are observed from signals that have little self-similarity and have temporal fluctuations that look more like random noise (Figure 1).

Recent evidence suggests that such variations in *H* measured with human neuroimaging characterize the overall availability of cognitive resources. In particular, lower values of *H* are observed during states in which more cognitive resources are engaged, that is, states that require more cognitive effort (Churchill et al., 2015; Kardan et al., 2019, 2023; Suckling et al., 2008; Wei et al., 2013). In addition, the degree to which *H* values decrease from baseline may indicate the degree of cognitive resources engaged (Churchill et al., 2015; Kardan et al., 2023): the highest values of *H* are observed at rest (Suckling et al., 2008) with lower values observed in easy cognitive tasks and lower values, still, observed in hard cognitive tasks (Churchill et al., 2015). Similarly, it was found that individuals performing a working memory task who suppress *H* more from baseline tend to engage more cognitive effort (Kardan et al., 2023).

One possible explanation for this convergence of findings is that *H* serves as a real-time index of how close the brain is to a critical state (Cocchi, Gollo, Zalesky, & Breakspear, 2017) (see Touboul & Destexhe, 2017, however, for a discussion of systems with high *H* in the absence of criticality), which serves as a proxy for the overall optimality of human neural networks and the degree to which they maximize energy use for neural activity (Roberts, Iyer, Vanhatalo, & Breakspear, 2014). The concept of critical states is an idea borrowed from statistical mechanics and the physics of complex systems which describes systems at points of transition. For example, water at 374 Celsius and 3,200 psi readily (i.e., with little energy) moves between a liquid, a gas, and a solid state. For complex networks, like the brain, critical states (with values of *H* close to 1) provide maximum dynamic range (Gautam, Hoang, McClanahan, Grady, & Shew, 2015; Kinouchi & Copelli, 2006) and optimized information storage and transfer (Boedecker, Obst, Lizier, Mayer, & Asada, 2012; Shriki et al., 2013; Shriki & Yellin, 2016). These findings suggest that, in part, measurements of *H* quantify the flexibility and efficiency of cognitive computations.

In addition, the connection between *H* and the theoretical framework of criticality provides a rich and well-developed set of mathematical tools from physics and complex systems that can allow empirical findings to rapidly inspire and inform concrete mathematical and mechanistic models of brain function and cognition. In contrast, while network science characterizations of brain states (Bassett & Sporns, 2017) are more widely applied than *H*, mathematical tools for understanding the emergence of network structure and dynamics have only recently begun to be developed (Ashourvan, Gu, Mattar, Vettel, & Bassett, 2017; Bassett et al., 2018; Gu et al., 2018; Zöller et al., 2021).

Consequently, individual differences in task *H* are of great interest to research on psychopathology: decreased cognitive resources (Medaglia, Pasqualetti, Hamilton, Thompson-Schill, & Bassett, 2017) and variations in the allocation of cognitive resources among different cognitive systems have been linked to depression, impulsivity, poor behavioral performance on cognitive tasks and have been linked to risk for essentially all forms of psychopathology (Jones, Siegle, Muelly, Haggerty, & Ghinassi, 2010; Levens, Muhtadie, & Gotlib, 2009, Moore et al., 2020).

How, though, might measurements of *H* lead to a better understanding of the relationship between psychopathology and associated cognitive impairment? In order to develop hypotheses about this relationship, we build on two previously mentioned findings within the *H* literature. First, decreased *H* is consistently observed under conditions of increased mental effort (e.g., during tasks [Churchill et al., 2015; He, 2011], with typical aging in adults [Churchill et al., 2015], and in psychopathology [Ide et al., 2016; Lai et al., 2010; Sokunbi, 2018; Sokunbi et al., 2014; Wei et al., 2013]).

Second, regional variation in the degree to which *H* is reduced (see Supporting Information Figure S1 for an example of absolute regional variation during a single task, the EN-Back task) during task performance (compared to rest) may be dependent on the specific demands of the task (Churchill et al., 2015; He, 2011). These observations suggest that changes in *H* may originate endogenously (e.g., with age or psychopathology) or exogenously (e.g., via task demands). Importantly, the significance of such changes in *H* vary depending on their source. For example, lower *H* in older individuals has been associated with decreased cognitive performance related to normal aging (Churchill et al., 2015). In contrast, in the context of a cognitive task, higher *H* (i.e., a failure to suppress *H*) might indicate a lack of engagement with the task (Kardan et al., 2023; Suckling et al., 2008; Zhuang et al., 2022) and poorer performance.

**Figure 2.**
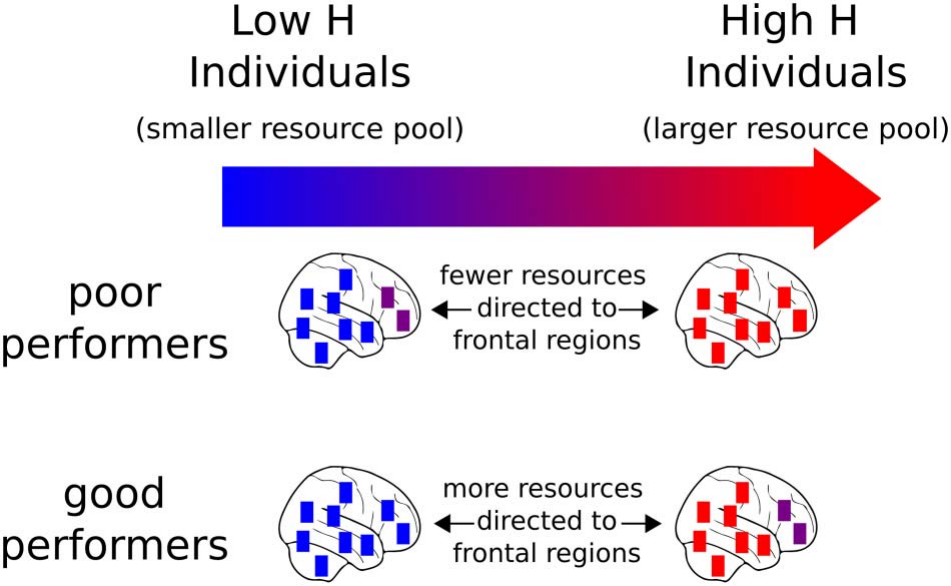
***Hypothesis 2***. While some individuals differ in overall, whole-brain patterns of *H* (see Figure 1), we expect relatively higher *H* in cognitive brain networks to be indicative of fewer resources directed toward the task at hand and poorer task performance, and vice versa. Here we represent this hypothesis by showing the distribution of *H* throughout the brain for individuals with both high *H* and low *H* performing a task that requires recruiting frontal regions. Relative to the whole brain tendency of high or low *H*, we hypothesize that individuals who perform poorly would have higher *H* in frontal regions. This indicates a failure to properly engage and direct cognitive resources to those regions. In contrast, we hypothesize that individuals who perform well would have lower *H* in frontal regions. This indicates that those individuals engage those regions and direct cognitive resources toward them, resulting in good performance on the task.

Consequently, we propose two hypotheses. Hypothesis 1: decreased *H* associated with psychopathology is indicative of reduced global cognitive resources and should be associated with poorer behavioral performance (Figure 1). Hypothesis 2: higher *H* within task-related cognitive brain networks is indicative of fewer cognitive resources allocated to the task and subsequent poorer performance (Figure 2). Importantly, Hypothesis 2 is made relative to an individual’s whole brain pattern of *H*; an individual may have lower *H* overall (e.g., due to psychopathology as in Hypothesis 1), but might still have higher *H* within working memory–related brain areas compared to sensorimotor brain areas, for example.

In summary, we hypothesized that global reductions in *H* would be related to increased psychopathology (i.e., fewer cognitive resources available), and worse overall behavioral performance. Second, we hypothesized a more nuanced effect of a psychopathology-associated increase in *H* in task-related areas (i.e., suboptimal resource allocation of available resources) related to poorer cognitive performance. Here, we present evidence for these hypotheses in a large diverse sample of children and in a replication sample of adults.

## RESULTS

In order to test our two hypotheses and establish a transdimensional relationship between *H* and psychopathology, we used fMRI time series from the emotional n-back working memory task (EN-Back) and scores on the parent-rated Child Behavior Checklist (CBCL) from the baseline year of the Adolescent Brain Cognitive Development^*SM*^ Study (ABCD) dataset (Release 2.0.1) (Hagler et al., 2019).

CBCL scores were fit to a bifactor model (Caspi et al., 2014; Kaczkurkin et al., 2020; Lahey et al., 2012; Lahey, Krueger, Rathouz, Waldman, & Zald, 2017; Reise, 2012) via confirmatory factor analysis (see Methods; Moore et al. [2020]). This model asserts that essentially every dimension of psychopathology is positively correlated because they all share causes and psychobiological mechanisms to a considerable degree (Lahey et al., 2017). Rather than studying the correlates of every form of psychopathology separately, the bifactor model specifies a general factor (also referred to as the p-factor [Caspi et al., 2014]) that reflects transdimensional processes contributing to the development of mental disorders in general. This model also specifies orthogonal specific factors that reflect processes that uniquely contribute to subsets of psychiatric disorders. Psychopathology was assessed via extracted factors scores from the bifactor model for each subject on the general factor and three specific factors: externalizing, internalizing, and ADHD.

We calculated *H* from each subject’s fMRI BOLD time series during runs of the EN-Back task, which measures recognition memory in the presence of varying emotional distractors (Hagler et al., 2019; Rosenberg et al., 2020). Briefly, participants saw a series of images during each block of the EN-Back task and were asked to indicate whether the current image matches the *n*th previous image. For example, 2-back blocks require remembering images that appeared two images before the current image, while 0-back blocks serve as a target detection task in which participants were shown a target image at the beginning of the block and were instructed to respond when the presented image matched the target (Rosenberg et al., 2020). Images consisted of either physical places or faces expressing happy, fearful, or neutral emotions. This task simultaneously probes emotional regulation and working memory, both of which have been implicated transdimensionally in childhood psychopathology (Harden et al., 2020; Huang-Pollock, Shapiro, Galloway-Long, & Weigard, 2017; Keenan, 2000; Reininghaus et al., 2019; Weissman et al., 2019; Wills, Simons, Sussman, & Knight, 2016). After preprocessing, visual quality control, and exclusion for high head motion, data from 1,839 children were retained. The EN-Back BOLD time series were parcellated into 392 previously defined cortical and subcortical parcels (Craddock, James, Holtzheimer, Hu, & Mayberg, 2012) and *H* for each parcel was then computed (see Methods).

While our primary interest was in childhood psychopathology, toward out-of-sample replication, we also analyzed data from 703 adults aged 25–35 from the Human Connectome Project dataset (HCP) (Glasser et al., 2013) (see Methods). *H* exponents were calculated from fMRI BOLD time series during a standard working memory n-back task. Psychopathology was assessed based on responses to the Achenbach Adult Self-Report (Achenbach & Rescorla, 2007; Barch et al., 2013). We note that this sample was recruited to have low levels of psychopathology (Van Essen et al., 2013).

### A Distributed Task-Induced Hurst-Psychopathology Pattern

**Figure 3.**
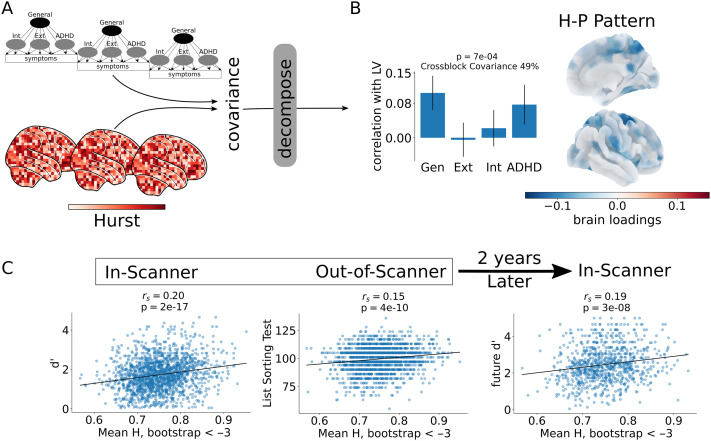
A Task-induced pattern relating scale-free brain activity as measured by *H* to childhood psychopathology. (A) The covariance matrix between *H* and extracted bifactor scores was decomposed with PLS to find maximally covarying latent-variables. (B) A single latent variable, which explained 49% of the cross-block covariance was significant. This LV describes a Hurst-psychopathology pattern in which lower *H* is associated with both higher general and ADHD extracted factor scores. (C) Mean *H* for brain regions with absolute value bootstrap ratios > 3 is positively correlated with both in-scanner (left) and out-of-scanner (middle) and future in-scanner (right) working memory performance (note: there were no regions with positive bootstrap ratios greater than this threshold). This indicates that individuals with lower *H* tend to have higher extracted general and ADHD bifactor scores and also worse working memory performance and that these deficits persist over time (years) and across tasks (the in-scanner and out-of-scanner memory tasks were different, i.e., EN-back task vs. the List Sorting working memory task).

We related *H* to extracted bifactor scores using partial least squares analysis (PLS). PLS is a multivariate technique that extracts maximally covarying latent variables from two sets of data (Krishnan, Williams, McIntosh, & Abdi, 2011; McIntosh & Lobaugh, 2004) (Figure 3A); in this case *H* in each brain parcel (set 1) with extracted bifactor psychopathology scores (set 2). These latent variables (LVs) consist of loadings on to each of the two sets of data that specify the contribution of variables (e.g., *H* in occipital cortex, or ADHD extracted bifactor scores) to the LV. Statistically, the LV as a whole is assessed for significance with permutation testing, and the influence of individual variables is assessed via bootstrap resampling. Importantly, this analysis allows for a direct, multivariate, association between the temporal dynamics of the BOLD signal, characterized by *H*, and psychopathology.

**Figure 4.**
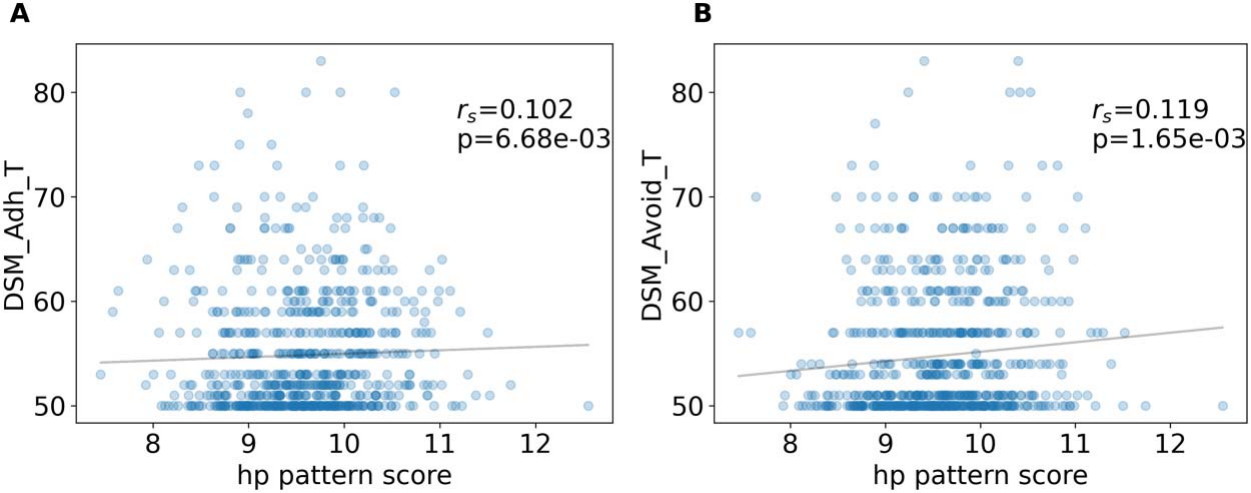
HPP scores in the HCP sample are significantly correlated with scores on the ASR DSM-Oriented Attention Deficit/Hyperactivity Problems and Avoidant Personality Problems scales. Spatial correlations between the HPP and each subject’s *H* map assess the degree to which each subject exemplifies the HPP. These were correlated with ASR DSM-Oriented scales and only significant correlations after correction for multiple comparisons across all six scales were retained.

This analysis revealed a single statistically significant latent variable relating *H* with childhood psychopathology (*p* = 0.0007, 10,000 permutations) that captures 49% of the covariance between *H* and extracted bifactor scores. This latent variable has stable positive loadings on the general and ADHD factors and stable negative loadings onto *H* in the brain, where stability was assessed with bootstrap ratios (Figure 3B, see Methods). Only brain areas with negative loadings were stable (absolute value bootstrap ratios > 3, see Supporting Information Table S8). This pattern of psychopathology loadings and brain loadings represents a distributed Hurst-psychopathology pattern (HPP) associating higher extracted bifactor scores (notably the general factor of psychopathology and ADHD) with lower *H*. In addition, the degree to which individuals in the adult HCP sample exemplified the HPP, which was defined solely in the ABCD dataset (see Methods), was significantly associated with computed scores on DSM-oriented scales for attention deficit and avoidance (Figure 4, Supporting Information Table S2), suggesting that the HPP maintains relevancy for psychopathology into adulthood—even in a population specifically recruited to have low levels of psychopathology—and beyond the narrow age range of the ABCD study sample (Dick et al., 2021; Heeringa & Berglund, 2020). Thus, a more detailed understanding of the developmental trajectory of brain-behavior relationship represented by the HPP is of particular interest for future studies, particularly those that analyze future longitudinal waves of ABCD data. These results support Hypothesis 1 and match the heuristic supported by past research of lower *H* being associated with more effort, where psychopathology is understood to be a more effortful state (in the colloquial sense).

While a large portion of the ABCD study sample was excluded due to poor quality or missing data, our subsample of 1,839 children was still close to nationally representative (Supporting Information Table S1). However, to increase our certainty that these associations would replicate beyond the characteristics of our subsample (e.g., socio-demographic factors or scanner manufacturer), we ran a number of sensitivity tests to account for representativeness (see Methods). These tests demonstrated that the HPP was robust to exclusion of multiple family members (randomly retaining only one participant per family), to conditioning on the acquisition site and scanner, to the application of poststratification weights (to account for differences between the full ABCD sample and nationally representative socio-demographics) and nonparticipation weights (to account for which children were able to complete the EN-Back task with low head motion), and to the use of CBCL syndrome scales in place of extracted bifactor scores; see Methods, Supporting Information Table S1, Supporting Information Figures S2 and S3. We additionally replicated these analyses in a k-fold cross-validation framework to ensure the generalizability of the HPP (i.e., that the HPP is not the result of overfitting; Supporting Information Figures S4 and S5). Finally, we replicated these results after censoring individual fMRI frames with high motion (framewise displacement > .2 mm), which has been shown in this sample (Fair et al., 2020) to reduce brain-behavior false positives (Supporting Information Figure S6).

### Relationship Between the HPP and Task Performance

Next, we asked whether the HPP was associated with working memory performance. In order to do so, we averaged *H* over the stable regions in the HPP, that is, the regions with absolute value bootstrap ratios > 3 (see Figure 3B). Task performance was assessed in-scanner by sensitivity, *d*′, and accuracy both during the 2-back blocks of the EN-Back task, and out-of-scanner by unadjusted, standardized scores on the List Sorting working memory task from the NIH cognitive toolbox (Weintraub et al., 2013). We note that while 2-back accuracy and *d*′ are highly correlated (*r*_*s*_ = 0.87, *p* = 0.0), accuracy in this context is strongly influenced by true negatives (correct rejections) while *d*′ better captures the balance between hits (true positives) and false alarms (false positives). Mean *H* in the HPP was significantly correlated (Spearman’s rank correlation coefficient, *r*_*s*_) to in-scanner performance (for *d*′, *r*_*s*_ = 0.20, *p* = 2*e* − 17; for acc, *r*_*s*_ = 0.25, *p* = 2*e* − 25) and out-of-scanner performance (*r*_*s*_ = 0.15, *p* = 4*e* − 10), Figure 3C, indicating that higher *H* is related to better performance both in-scanner and out-of-scanner. Mean *H* in the HPP was also significantly related to out-of-scanner performance when controlling for in-scanner performance (Supporting Information Table S3), suggesting that the HPP is related to working memory ability in a trait-like manner. This is further supported by the fact that mean *H* in the HPP was significantly correlated with working memory performance in the adult HCP sample (*r*_*s*_ = 0.18, *p* = 2*e* − 6). These results are in line with Hypothesis 1 that decreased *H* associated transdimensionally with psychopathology is indicative of reduced global cognitive resources (Figure 1).

Additionally, a mediation analysis (see Methods) indicated that mean *H* in the HPP partially mediates the relationship between extracted bifactor scores and in-scanner working memory performance (Supporting Information Table S6). When extracted bifactor scores are treated as the mediator instead of *H* (Supporting Information Table S7), the significant paths remain the same, however, there is no longer a strong relationship between extracted bifactor scores and 2-back accuracy. Thus, while there is no statistical preference for *H* over extracted bifactor scores as a mediator, we can conclude that *H* has a more direct relationship with 2-back working memory performance than psychopathology.

Next, we assessed the relationship between mean *H* in the HPP and future in-scanner 2-back *d*′ and accuracy during the 2-year follow-up session. Specifically, we correlated the same baseline HPP measure for each participant with in-scanner 2-back accuracy and *d*′ during the 2-year follow-up session for participants that had data for both sessions (*N* = 888, approximately half of the full sample had 2-year follow-up data available in ABCD Release 3.0). Since the sample of individuals who had available data for ABCD Release 3.0 is nonrandom (see Supporting Information Table S4), we additionally computed weighted correlations to correct for nonparticipation (see Methods). Only future *d*′ was significantly correlated with mean *H* in the HPP after correction for nonparticipation (*r*_*s*_ = 0.19, *p* = 3*e* − 8; $r_{s}^{\text{corrected}}$ = 0.089, *p*^*corrected*^ = 0.008, Figure 3C). In addition, though baseline in-scanner 2-back *d*′ and 2-year follow-up in-scanner 2-back *d*′ are significantly correlated (*r*_*s*_ = 0.51, *p* = 4*e* − 57), mean *H* in the HPP was significantly related to future *d*′ when controlling for baseline *d*′ (Supporting Information Table S5).

In summary, these results support Hypothesis 1 and indicate that the HPP is associated with trait-like deficits in working memory that persist over long time periods (i.e., years), are seen in at least two distinct working memory tasks, and are relevant to working memory performance in adults.

### The HPP Defines a Functional Activation Axis

We next sought to better understand the meaning of the HPP and to understand its context in the larger body of literature investigating functional activation, task performance, and psychopathology. To do so, we determined whether there were patterns of functional activation within large-scale cognitive systems associated with the HPP.

**Figure 5.**
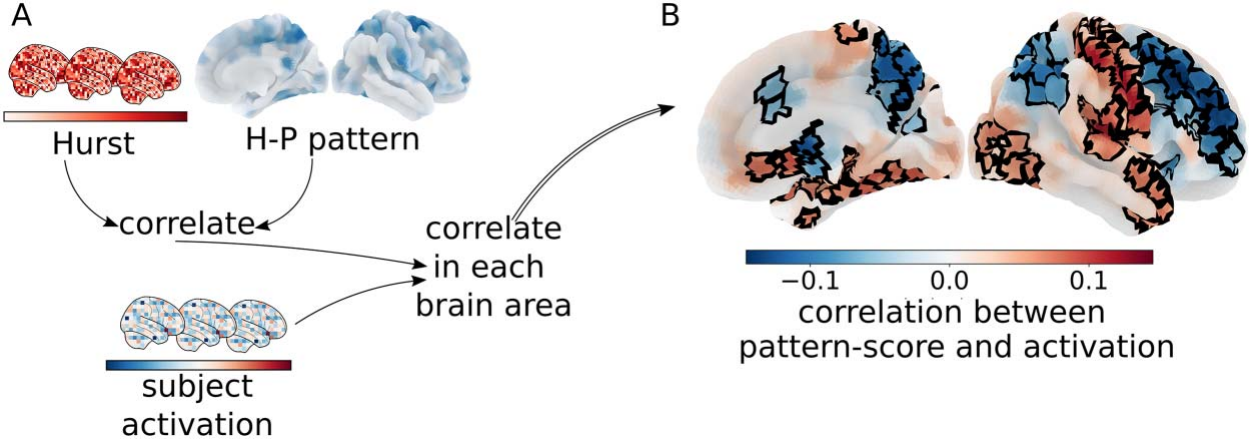
The Hurst-psychopathology pattern defines a functional activation axis associated with task performance and psychopathology. (A) To understand the relationship between the HPP and functional activation we first computed a pattern score for each subject by correlating their spatial patterns of *H* to the HPP. Next, we correlated these pattern scores across participants to functional activation in each brain parcel. (B) Higher general factor of psychopathology and ADHD extracted factor scores and lower *H* are associated with decreased fronto-parietal activation and increased occipital, medial-temporal, and sensorimotor activation on 2-back versus 0-back blocks. Areas where pattern scores are significantly correlated with functional activation after multiple comparison correction are outlined in black.

We examined the relationship between individual participants’ Hurst-psychopathology associations and their own simultaneous functional activation (i.e., BOLD signal contrast during the same run). Specifically, we asked how each subject’s pattern of activations during the EN-Back task was related to the degree to which they exemplify the HPP. To do so, we generated a pattern score for each subject by correlating their Hurst map with the HPP map. Thus, the pattern scores represent the spatial similarity between individual participants’ Hurst maps and the HPP map. This pattern score quantifies how much each participant expresses the HPP map. Higher pattern scores are statistically indicative of higher levels of psychopathology, lower *H*, and poorer task/cognitive performance. Next, activations were defined as the contrast between 2-back and 0-back blocks in 148 cortical regions (see Figure 5A and Methods; Hagler et al., 2019), we additionally examined the contrast between emotional and neutral faces, positive and neutral faces, and negative and neutral faces, but there were no significant associations for these contrasts with the HPP after multiple comparison correction; Supporting Information Figures S7–S9). Finally, we correlated the activation values and subject pattern scores in each of the 148 cortical regions. A positive correlation implies that increased activation (2-back vs. 0-back contrast) is associated with higher extracted bifactor scores, and a negative correlation implies that decreased activation is associated with higher extracted bifactor scores.

The resulting map (Figure 5B) indicates that higher pattern scores, (i.e., higher general and ADHD extracted bifactor scores and lower *H*), and poorer working memory performance are associated with decreased fronto-parietal activation and increased sensorimotor activation in the same task. This is in line with previous work in this sample (Rosenberg et al., 2020), which found that lower performance was associated with decreased functional activation (2-back vs. 0-back) in fronto-parietal areas commonly associated with cognitive processes related to this working memory and emotional regulation task. This convergence of findings suggests overlap among the mechanisms driving poorer task performance generally, and the mechanisms driving psychopathology specifically. In other words, this pattern of functional activation—which represents a fronto-parietal/sensorimotor axis of resource use—is not specific to psychopathology. However, its association with psychopathology and *H* here is consistent with Hypothesis 2 and suggests that the general factor of psychopathology and the ADHD specific factor are associated with a relative redistribution of cognitive resources away from task-relevant brain networks and into sensorimotor processing.

### The HPP Defines a Psychological Axis

Next, we sought to understand the significance of spatial variation in the strength of the HPP (i.e., the relationship between Hurst exponents and psychopathology) across different brain regions. For example, this relationship is stronger in some parts of insular cortex and in middle frontal gyrus (two areas associated with emotional regulation and working memory, respectively), but weaker in some parts of occipital cortex and anterior frontal cortex (Supporting Information Table S8). Consequently, we sought to better understand the psychological and cognitive significance of these spatial variations within the HPP. In other words, we sought to characterize the data-derived HPP in reference to the broader neuroimaging literature independent of the operationalizations of the present study to increase interpretability.

For this analysis, we first examined similarities between the HPP and probabilistic meta-analysis maps from Neurosynth (Yarkoni, Poldrack, Nichols, Van Essen, & Wager, 2011) that describe how frequently journal articles contain specific terms alongside voxel coordinates related to functional activation. Thus, these terms capture, broadly, the different instantiations of brain activation for cognitive constructs in specific tasks used by individual studies. In other words, Neurosynth provides the common brain activation patterns across studies for different cognitive constructs, such as spatial attention, rehearsal, and effort. We expected this analysis to reveal a psychological axis defined by the HPP that contrasts psychological terms which are similar and dissimilar to the HPP.

**Figure 6.**
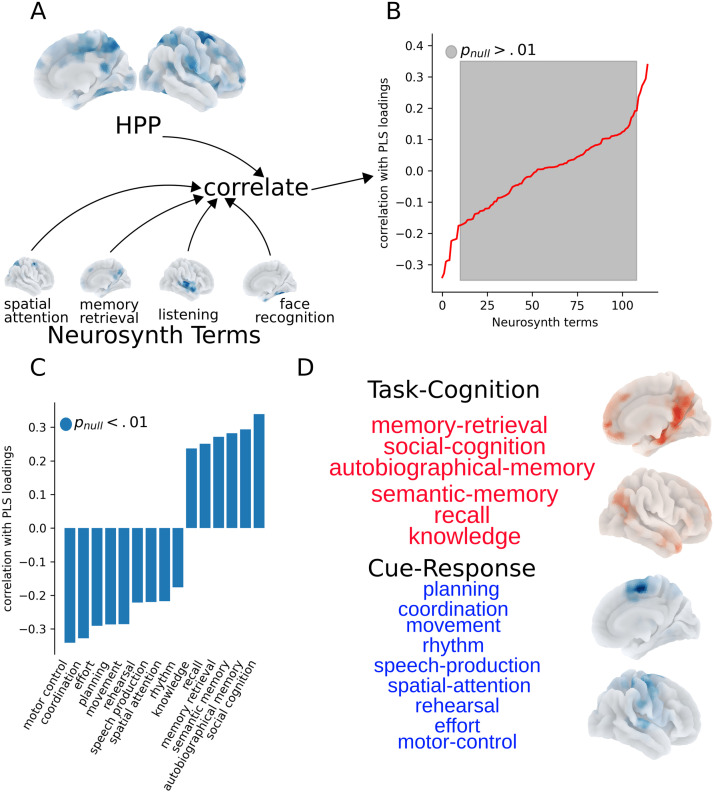
The Hurst-psychopathology pattern defines a psychological axis associated with task performance and psychopathology. (A) We correlated the HPP with 116 Neurosynth term association maps showing the probability of activation with multiple psychological terms (shown for the terms “spatial attention,” “memory retrieval,” “listening,” and “face recognition”) to identify which terms had spatial patterns of activation most similar to the HPP. (B) Gray indicates nonsignificance based on 1,000 parametric spatial permutation tests (Benjamini-Hochberg correction, *α* = .01). On the *x*-axis, terms are ranked by the magnitude and sign of correlations. (C) Terms that are positively correlated with the HPP are the positive term set and terms that are negatively correlated with the HPP are the negative term set. (D) The positively correlated terms include task-relevant cognitive processes. The negatively correlated terms included processes involved in planning and executing responses to task cues. Surface maps of these two sets were created by taking the maximum value across term maps included in each set.

We used a previously defined subset of 125 terms (Hansen et al., 2021) which included, for example, “cognitive control,” “language comprehension,” “memory,” “psychosis,” and “social cognition.” This term set was restricted to the overlap between Neurosynth terms and Cognitive Atlas (Poldrack et al., 2011) terms and can thus be thought of as belonging to a proposed classification of psychological concepts and tasks. Each term’s associated activation map was correlated with the HPP, and significance was assessed via a spatial null model (see Methods and Figure 6A). Importantly, these Neurosynth maps only indicate which regions are commonly reported alongside a given term and do not address the sign of the association (i.e., whether the psychological term is associated with functional activation or deactivation).

After correction for multiple comparisons, 15 terms remained (Figure 6B and C). The set of terms positively correlated to the HPP define a Task-Cognition category, which includes terms related to the cognitive processes engaged by the EN-Back task (Rosenberg et al., 2020): emotional regulation and working memory (Figure 6C and D, top). The set of terms negatively correlated to the HPP define a Cue-Response category, which includes terms related to processes involved in planning and executing responses to the task cues (Figure 6C and D, bottom). These results describe a Task-Cognition/Cue-Response axis (see Supporting Information text for an in-depth description of the term sets) of cognitive function that is relevant to childhood psychopathology. Further, in support of Hypothesis 2, these results suggest that the HPP is associated with a functional trade-off between Task-Cognition and Cue-Response areas.

### Relative Differences in *H* Between Cognitive Networks

Next, to further evaluate Hypothesis 2, we sought to test whether Task-Cognition and Cue-Response areas showed differences in *H* relative to psychopathology and individuals whole-brain patterns of higher or lower *H*. To do so, we took the union across term maps in the Task-Cognition and Cue-Response sets by choosing the maximum z-score across term maps in each brain region. For example, if “semantic-memory” and “knowledge” had z-scores of +2 and +3 in a portion of the medial prefrontal cortex, we assigned that region a z-score of +3 in the combined map. This resulted in combined maps that are maximally representative of the Task-Cognition and Cue-Response term sets. Next, we kept regions above a z-score threshold and calculated the difference between the mean *H* for Task-Cognition and Cue-Response. Finally, we correlated this difference across participants to pattern scores (i.e., the degree to which each subject exemplifies the HPP during the EN-back task). This resulted in a significant positive correlation across all choices of z-score threshold (Supporting Information Figure S10). Since higher pattern scores are statistically indicative of higher psychopathology and poorer working memory performance, this result is consistent with the first part of Hypothesis 2 and indicates that psychopathology is associated with increases in *H* in Task-Cognition areas and decreases in *H* in Cue-Response areas, relative to individuals’ own whole-brain *H*.

Further, this relative difference in *H* between Task-Cognition and Cue-Response areas tends to be exacerbated in individuals with higher general and ADHD extracted bifactor scores, as demonstrated by positive correlations in Supporting Information Figure S10. This parallels the functional fronto-parietal/sensorimotor axis, which indicated that psychopathology is associated with reduced functional activation in fronto-parietal areas and increased functional activation in sensorimotor areas.

More specifically, the fronto-parietal/sensorimotor functional axis defined by the HPP identifies two networks of brain areas in which reduced/increased functional activation is associated with psychopathology. These areas (Figure 5) broadly fit into two groups that are associated with task-related and sensorimotor processes, respectively. Similarly, the Task-Cognition/Cue-Response psychological axis defined by the HPP identifies two sets of psychological terms (Figure 6) that are associated with similar sets of brain areas as are identified by the functional axis.

The existence of these two parallel psychological and functional axes associated with the HPP is consistent with Hypothesis 2 and suggests that psychopathology is associated with a reduction in resource allocation to Task-Cognition areas, relative to Cue-Response areas. In other words, individuals with higher levels of psychopathology tend to engage relatively fewer fronto-parietal resources relevant to the emotional regulation and working memory demands of the EN-Back task. They also tend to engage relatively more sensorimotor resources relevant to the rote sensory and motor demands of the task.

### Associations of the HPP With Block Level Hurst Exponents

The previous analyses implicitly contrasted Task-Cognition/fronto-parietal (cognitive) and Cue-Response/sensorimotor areas and suggested that psychopathology is associated with a relative redistribution of resources away from cognitive regions, relative to sensorimotor regions. However, those analyses did not establish whether cognitive regions or sensorimotor regions drive this relative redistribution of resources associated with psychopathology. Thus, we sought to determine whether a net decrease in resources allocated toward cognitive regions, a net increase in resources allocated toward sensorimotor regions, or a combination of both, was responsible for this effect. In other words, we sought to better understand which brain regions drive the relative redistribution of resources away from cognitive regions seen in the previous analyses.

To this end, we calculated *H* for 2-back and 0-back blocks of the EN-Back task separately and then averaged *H* across all blocks of the same type for each subject (see Methods; Kardan et al., 2022). We averaged *H* within the cognitive and sensorimotor regions at various z-score thresholds on the Neurosynth maps and subtracted 0-back average cognitive and sensorimotor *H* from 2-back average cognitive and sensorimotor *H*, respectively. This created a contrast between 2-back and 0-back blocks within cognitive and sensorimotor areas that would indicate the degree to which *H* was suppressed (Churchill et al., 2015) during 2-back blocks, relative to 0-back blocks. Finally, we correlated this contrast with 2-back accuracy (acc), sensitivity (*d*′), and participant HPP scores.

As expected, irrespective of psychopathology, suppression of *H* during 2-back in cognitive and sensorimotor areas (i.e., lower Hurst during 2-back compared to 0-back) was significantly associated with superior performance as measured by *d*′ (Supporting Information Figure S12; for sensorimotor regions *p* < .05 for Neurosynth z-score threshold, z < 3.44; for cognitive regions *p* < .05 for z < 1.85). This effect was driven by hit rate (the percent of responses that are correct) rather than the false alarm rate (Supporting Information Figure S13). However, only decreased *H* during 2-back in the cognitive regions were significantly associated with 2-back accuracy (Supporting Information Figure S14, for cognitive regions *p* < .05 for z < 1.61). These observations further support Hypothesis 2 that relative to individuals’ own whole-brain patterns of *H*, higher *H* within cognitive brain networks are indicative of fewer cognitive resources allocated toward those cognitive systems, and vice versa.

**Figure 7.**
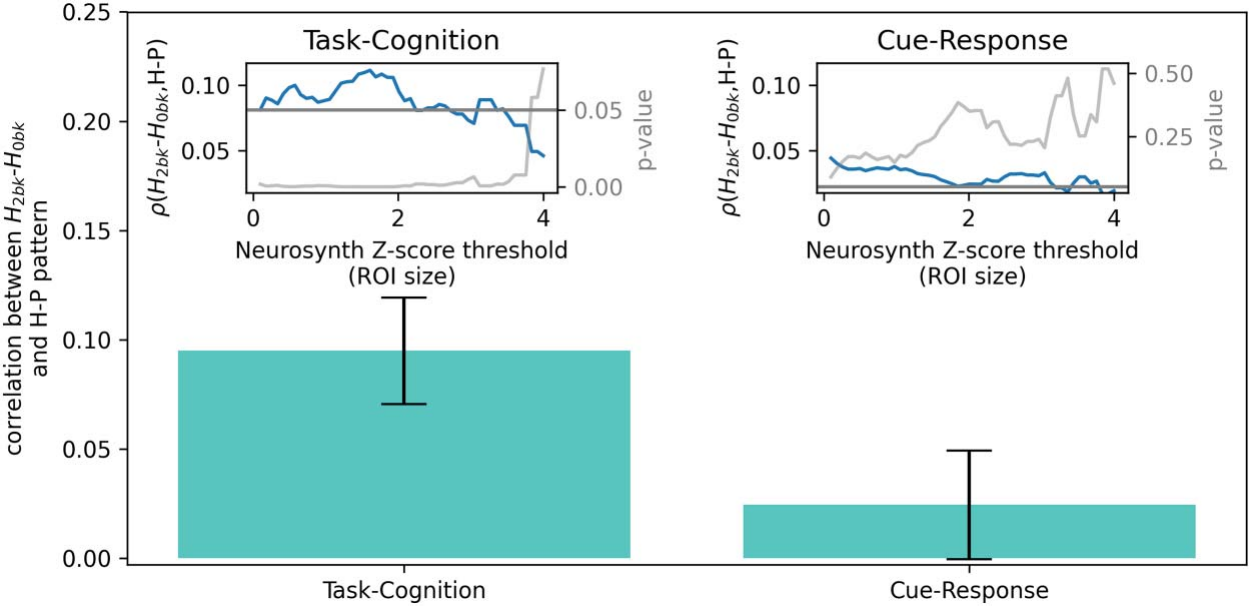
Correlations (blue) were computed across all choices of z-score threshold used to define the task-cognition and cue-response ROIs from Neurosynth meta-analysis probabilistic activation maps. The significance of the correlations was assessed at all choices of threshold (gray). The *p* = 0.05 level is shown as the gray horizontal line. We found evidence of a correlation between higher *H* and higher HPP scores only for task-cognition areas. Insets show correlations between block level 2- versus 0-back *H* contrast and HPP scores for all choices of z-score threshold. The bar plot shows the correlation for the choice of z = 2. Error bars represent standard deviations from 1,000 bootstrap resamples.

In contrast, psychopathology, assessed via higher HPP scores, was significantly associated with less suppression of *H* during 2-back (i.e., less decrease in *H* during 2-back relative to 0-back) in cognitive areas, but not in sensorimotor areas (Figure 7; for cognitive regions *p* < .05 for z < 3.76). This indicates that psychopathology is related to less engagement of cognitive regions. Thus, while better task performance generally was associated with suppression of *H* in both cognitive and sensorimotor regions, we only found evidence that psychopathology and associated poorer task performance was associated with higher *H* in cognitive regions (i.e., less suppression of *H*; see Figure 2). This combination of results supports Hypothesis 2 and suggests that decreased resource allocation toward Task-Cognition/fronto-parietal (cognitive) regions alone drives the relative redistribution of resources away from cognitive regions associated with psychopathology. In other words, the observed relative difference in resource use between cognitive and sensorimotor regions results from failure to engage cognitive regions properly during the working memory-intensive task blocks.

## DISCUSSION

Building on previous evidence associated lower *H* with psychopathology and that *H* may quantify cognitive resource utilization during tasks, we hypothesized that measurements of *H* can provide mechanistic insight—at the functional phenomenology level of analysis (Bassett et al., 2018)—into the relationship between psychopathology and cognitive performance. Specifically, we hypothesized that (1) lower global *H* associated with psychopathology signals reduced cognitive resource availability and (2) that higher *H* localized within task-related brain networks signals fewer resources allocated to the task at hand.

As expected, we found a distributed multivariate pattern associating decreased *H* with increased extracted bifactor scores for the general factor of psychopathology and specific ADHD factor, thereby demonstrating a general, nonspecific association between *H* and psychopathology. Previous research has demonstrated associations between the *H* and specific mental disorders, primarily in small populations (Cerquera, Arns, Buitrago, Gutiérrez, & Freund, 2012; Ide et al., 2016; Lai et al., 2010; Long et al., 2018; Sokunbi, 2018; Sokunbi et al., 2014; Wei et al., 2013), but did not clarify whether the Hurst exponent was also associated transdimensionally with psychopathology in large diverse samples. In support of Hypothesis 1, we found that this pattern was associated with poorer task performance on multiple concurrent working memory tasks and with performance on the same working memory task 2 years later.

While the overall pattern observed was that of decreased *H* in individuals with higher extracted general factor and ADHD bifactor scores, there was spatial variation in the strength of this pattern across the brain that had not been previously studied. Toward Hypothesis 2 and to better understand the cognitive and psychological relevance of these spatial variations, we compared this pattern to simultaneous functional activation in the same individuals (Hagler et al., 2019) and to meta-analysis-derived activation maps associated with psychological and cognitive terms (Hansen et al., 2021; Shafiei et al., 2020; Yarkoni et al., 2011).

These analyses revealed that the pattern associating *H* and psychopathology defines a Cue-Response/Task-Cognition psychological axis and a parallel sensorimotor/fronto-parietal activation axis. These axes suggest that individuals with higher extracted bifactor scores tend to allocate fewer resources to Task-Cognition/fronto-parietal areas, measured via reduced functional activation (i.e., BOLD contrast) and relatively higher *H*. In addition, block-level analyses suggested that this effect was driven solely by a reduction in resources (measured via higher Hurst exponents) to Task-Cognition/fronto-parietal areas, in line with Hypothesis 2.

Under the theoretical framework of criticality in which *H* is a candidate measure of cognitive resource allocation, these results suggest a mechanistic account of reduced task performance associated with psychopathology. Specifically, that psychopathology, in general, is associated with an overall reduction in cognitive resources and, importantly, with fewer resources directed toward task-specific cognition, and subsequent poorer task performance. Interestingly, this also suggests that Cue-Response/sensorimotor areas may have priority when it comes to resource allocation.

Importantly, this proposed mechanistic account is fairly coarse-grained (Bassett et al., 2018) (i.e., concerned with large, distributed brain networks as opposed to individual brain areas) and speaks to functional phenomenology in the brain (Bassett et al., 2018), that is, statistical and temporal trends in brain activity, as opposed to specific cellular and electrophysiological dynamics. Thus, it is important that the results presented here are primarily interpreted within this context and that potential implications for mechanisms at other levels of analysis (e.g., cellular or behavioral) should be carefully and thoroughly investigated. In addition, we note that future studies with quasi-experimental or panel data, such as future longitudinal releases of the ABCD study, are required for more definite causal evidence to support the proposed mechanistic account of reduced task performance associated with psychopathology.

In addition, the results presented here are in line with the recent evidence that *H* is a quantitative measure of available cognitive resources and/or mental effort (Churchill et al., 2015; He, 2011; Kardan et al., 2023; Suckling et al., 2008). Under this proposal, deviations from a theoretical state of “perfect” rest, which is assumed to be organized near a critical state and to have a Hurst exponent of 1 (de Arcangelis, Perrone-Capano, & Herrmann, 2006; Kitzbichler, Smith, Christensen, & Bullmore, 2009), are indicative of decreased cognitive resources and/or increased mental effort. As the demand for additional cognitive resources increases due to task demands, fatigue, or psychopathology, the brain moves further away from the critical state, reducing the available supply of flexible information processing resources in exchange for more context-relevant processing (Cocchi et al., 2017).

One of the primary limitations of these analyses is the potential for multiple temporal scaling regimes in the BOLD signal (Kantelhardt et al., 2002; Robinson, 2003; Wendt, Roux, Jaffard, & Abry, 2009). The detrended fluctuation analysis (DFA) method of estimating *H*, used here, assumes a “mono-fractal” process with a single scaling regime. DFA tends to be superior to other methods, such as power spectral density (PSD), that are more sensitive to nonstationarities in the signals (Churchill et al., 2015). Examination of the goodness of fit for DFA regression line revealed that for the most part, the data in this study are captured by a “mono-fractal” process (Supporting Information Figure S11). However, these fits are not perfect and there may be a small benefit to employing other methods for calculating *H* which can capture multiple scaling regimes (Kantelhardt et al., 2002; Wendt et al., 2009).

Additionally, the influence of respiratory artifacts on brain-behavior relationships in fMRI data has recently been of interest (Fair et al., 2020). While we did not directly control for the potential presence of respiratory artifacts in this study, our results were robust to stringent motion censoring (removal) of frames with framewise displacement > .2 mm, which has been shown, in the same ABCD sample, to be highly effective in reducing respiratory-related brain-behavior false positives (Kay et al., 2022). Similarly, the consistency among the between-subject analyses (sections A Distributed Task-Induced Hurst-Psychopathology Pattern and Relative Differences in *H* Between Cognitive Networks) and the within-subject analyses (section Associations of the HPP With Block Level Hurst Exponents) suggest that respiratory artifacts are not primarily driving the results, though they could still contribute a small effect.

Previous research has suggested that lower *H* is characteristic of harder tasks (Churchill et al., 2015) and more difficult versions of the same task (Churchill et al., 2015; Kardan et al., 2023; Zhuang et al., 2022), but has not clarified how Hurst exponents are related to perceived mental effort when exogenous task demands remain constant. If the results presented here were indicative of transient increases in mental effort, we would not expect generalization to different tasks or across time (i.e., to the same task being performed two years after brain activity was measured). However, we found that the Hurst-psychopathology pattern is predictive of out-of-scanner (a different task) and future (the same task 2 years later) working memory performance even when controlling for in-scanner and baseline performance, respectively. Thus, the association between the described Hurst-psychopathology pattern and working memory performance is relatively stable across time and across at least two working memory tasks as a scale-free neural signature of psychopathology. However, these results do not, on the whole, clarify whether deficits in cognitive resources precede or follow psychopathology or clarify the time periods over which decreases in available cognitive resources persist. Future work, possibly with the forthcoming longitudinal waves of the ABCD study dataset, might better be able to answer both questions.

The analysis of temporal fractals found in human neuroimaging data promises a better understanding of human cognition. Quantification of these fractals via the Hurst exponent provides an ingress to the well-developed literature of criticality and its models and mathematics that may help build concrete, mechanistic models of cognition and allow for systematic characterizations of brain states. As demonstrated here, working within the framework of criticality can engender novel mechanistic hypotheses of individual differences in cognition that may help specify concrete mathematical models of cognition. The need for such models (Bassett et al., 2018; Jolly & Chang, 2019) and mechanistic accounts of cognition has been an increasingly important goal in the cognitive sciences. We see the theoretical framework of criticality, and measurements of *H*, as adjacent to other contemporary frameworks (Ashourvan et al., 2017; Gu et al., 2017) that together may provide a comprehensive framework for understanding cognition. As such, continued exploration of the associations between *H* and cognition may help to elucidate what optimal brain states represent and how deviations from those brain states arise due to psychopathology.

## MATERIALS AND METHODS

### Adolescent Brain and Cognitive Development Data

#### Data and preprocessing.

We performed functional MRI preprocessing on ABCD study baseline year emotional N-Back data, which included 10,240 participants. Participants who were scanned on Phillips brand scanners were excluded because of a known error in the phase encoding direction while converting from DICOM to NIFTI format. We downloaded minimally processed structural and functional MRI scans from the ABCD data portal (https://nda.nih.gov/abcd). Minimal preprocessing included motion correction, B0 distortion correction, gradient warping correction, and resampling to an isotropic space (Hagler et al., 2019). Minimally processed data were preprocessed with a custom version of FMRIPREP (Esteban et al., 2019), a Nipype (Gorgolewski et al., 2011) based tool. Each participant’s structural T1w scan was first defaced with pydeface. Each T1w (T1-weighted) volume was then corrected for INU (intensity nonuniformity) using N4BiasFieldCorrection v2.1.0 (Tustison et al., 2010) and had been previously skull-stripped. Spatial normalization to the MNI152 nonlinear sixth generation template, the standard MNI template included with FSL, was performed through nonlinear registration with the antsRegistration tool of ANTs v2.1.0 (Avants, Epstein, Grossman, & Gee, 2008), using brain-extracted versions of both T1w volume and template. Brain tissue segmentation of cerebrospinal fluid (CSF), white matter (WM) and gray matter (GM) was performed on the brain-extracted T1w using fast (FSL v5.0.9; Zhang, Brady, & Smith, 2001). Functional data coregistered to the corresponding T1w anatomical image using boundary-based registration (Greve & Fischl, 2009) with six degrees of freedom, using flirt (FSL). Motion correcting transformations (based on motion parameters obtained from the minimally processed data), BOLD-to-T1w transformation, and T1w-to-template (MNI) warp were concatenated and applied in a single step using antsApplyTransforms (ANTs v2.1.0) using Lanczos interpolation. Physiological noise regressors were extracted and applied from tissue masks, and framewise displacement (Power et al., 2014) was calculated for each functional run using the implementation of Nipype. For more details of the pipeline see https://fmriprep.readthedocs.io.

Following Power, Barnes, Snyder, Schlaggar, and Petersen (2012) and Power et al. (2014), we performed a 36 parameter confound regression that included the time courses of mean CSF signal, mean global signal, mean WM signal, the six standard affine motion parameters (x, y, z, pitch, roll, and yaw), their squares, their derivatives, and the squared derivatives of these signals. We also simultaneously regressed out linear and quadratic trends to remove drift related signals. This was followed by the application of a band-pass filter with a high-pass cutoff of .008 Hz and a low-pass cutoff of .12 Hz via the 3dBandpass command in AFNI (Cox, 1996). The cleaned volumetric BOLD images were spatially averaged into the 392 parcel Craddock atlas (Craddock et al., 2012). Finally, for Siemens scanners, the first eight volumes were removed because they were used as the multiband reference. For GE scanners running DV25 software, five volumes were removed because the first 12 volumes were used as the multiband reference and then combined into a single volume and saved as the initial TR (leaving a total of five frames to be discarded). For GE scanners running DV26 software, 16 volumes were removed (Rosenberg et al., 2020). Runs included 362 whole-brain volumes after these discarded acquisitions.

Finally, all structural and functional scans were visually inspected to screen for scanner abnormalities, and to assess the accuracy of the registration and tissue segmentation processes. Only participants with passing structural scans and at least one passing functional scan were included for further analyses.

#### Cognitive task procedures.

The emotional n-back task engages processes related to memory and emotion regulation (Rosenberg et al., 2020). Each session consists of two approximately 5-minute fMRI runs in which participants complete four 0-back (low working memory load) and four 2-back (high working memory load) task blocks. Each task block consists of four types of stimuli: happy, fearful, and neutral face photographs, and place photographs (Rosenberg et al., 2020). Performance was quantified as percent accuracy and sensitivity, *d*′, on 2-back blocks (Kardan et al., 2022; Rosenberg et al., 2020). Sensitivity, *d*′, was computed as

$$-\sqrt{2}*\mathrm{erf}c^{-1}\left(2*\mathrm{hit}\right)+\sqrt{2}*\mathrm{erf}c^{-1}\left(2*fa\right)$$ (1)

where *erfc* is the complementary error function, *hit* is the hit rate in 2-back blocks, and *fa* is the false alarm rate in 2-back blocks.

### Human Connectome Project

#### fMRI data and preprocessing.

We analyzed data from the Human Connectome Project (HCP), a multisite consortium that collected MRI, behavioral, and demographic data from 1,022 participants. Minimally preprocessed, open-access n-back fMRI data were downloaded from connectomeDB (https://db.humanconnectome.org/) via Amazon Web Services. The acquisition parameters and prepossessing of these data have been described in detail elsewhere (Glasser et al., 2013). Briefly, this preprocessing included distortion correction, realignment, and transformation to a standard space. In addition, we a high-pass filter of 0.001 Hz via fslmaths (Jenkinson, Beckmann, Behrens, Woolrich, & Smith, 2012), and ICA-FIX denoising using the HCPpipelines (https://github.com/Washington-University/HCPpipelines) tool, which regresses out nuisance noise components effectively, similar to regressing out motion parameters and tissue type regressors (Parkes, Fulcher, Yücel, & Fornito, 2018), high-pass filtering. We retained participants with both the left-to-right and right-to-left phase encoding scans available for the working memory task. The scans from both runs were averaged and then time series within 392 previously defined functional parcels (Craddock et al., 2012) were extracted.

#### Cognitive task procedures.

We analyzed fMRI data from the n-back task runs. This task is designed to probe working memory performance (Barch et al., 2013). Briefly, each run consisted of 2-back blocks where participants respond whenever the current stimulus is the same as the one 2-back and 0-back blocks where a target cue is presented at the start of each block, and the subject must respond to any presentation of that stimulus during the block. Each run contained eight task blocks and four fixation blocks counterbalanced between the memory load conditions.

### Exclusion of Data

After preprocessing, all fMRI BOLD time courses were spatially averaged within 392 previously defined functional regions (Craddock et al., 2012). Individual runs with greater than .2 mm mean and 2 mm max framewise displacement were excluded, which when combined with visual quality inspections resulted in the retention of 1,839 participants from the ABCD study sample and 703 participants from the HCP sample. For each ABCD subject, if more than one run was retained, the parcellated time series were averaged over the two runs.

### Estimation of Hurst Exponents

We measured *H* of the mean BOLD time series of each of the 392 previously defined regions using detrended fluctuation analysis (DFA). This is a computationally efficient estimator of the Hurst exponent that is a more robust alternative to power-spectral-density-based methods and has been shown to exhibit convergence with more sophisticated estimators of *H* in fMRI data (Churchill et al., 2015). Briefly, DFA involves transforming a detrended time series, *X*(*t*), into an unbounded random walk, *X*(*t*) = ∑(*x*(*i*) − $\bar{x}$), and then calculating local linear fits to this random walk, *Y*(*t*), for various window sizes *n*. The root-mean-square fluctuations from the local linear trend is then calculated for each window size as *F*(*n*) = $\sqrt{\sum {\left(X\left(t\right)-Y\left(t\right)\right)}^{2}/N}$, where *N* is the number of windows of size *n*. Finally, *H* is calculated as the slope of a linear fit of *log*(*n*) vs. *log*(*F*(*n*)) (Churchill et al., 2016; Peng et al., 1994).

For “mono-fractal” processes, a single exponent *H* fully describes the scaling relationship between local fluctuations and window size. However, in real data, *H* may change as a function of the window size due to the presence of different scaling regimes, or trends in the data that are not controlled by the detrending step of DFA. Thus, it is critical to examine the goodness of fit for the DFA regression line to determine whether such confounds are present (Churchill et al., 2016). We examined the goodness of fit using the coefficient of determination, *R*^2^ (Supporting Information Figure S11).

We chose to use window sizes, *n*, which exclude possible low-frequency confounds below 0.01 Hz and high-frequency confounds above 0.1 Hz. We sampled the number of windows, *N*, approximately uniformly given this frequency constraint. For each number of windows, *N*, we chose the maximum window size *n* such that *n* · *N* was less than or equal to the number of time points in the data.

Since the periodic structure of the EN-back task could impose structure and order at the block level to the BOLD time series, we additionally computed *H* from a task-regressed time series. We regressed out the task block structure from the BOLD time series after convolving it with the canonical statistical parametric mapping (SPM) hemodynamic response function (hrf), along with the derivative and dispersion parameters of the hrf. After calculating *H* we ran the PLS analysis again on these task-regressed *H* values. Comparisons to the PLS results without regressing task structure revealed a high correlation between the brain loadings (i.e., the HPP pattern) that was significant with respect to spin nulls (Markello & Misic, 2021) (Supporting Information Figure S15, left). Additionally, the design score pattern was highly similar between the original PLS and the task-regressed PLS (Supporting Information Figure S15, right). This suggests that the task structure is not responsible for the results observed here. These analyses were performed in Python using the nilearn package.

### Psychopathology Measures

#### CBCL scales.

CBCL scales were retrieved from ABCD Release 2.0.1 tabulated data available on the NIMH Data Archive (https://nda.nih.gov/abcd). Calculated t-scores for each scale (Barch et al., 2018) were retrieved from the *abcd_cbcls01.txt* file. Definitions of the variable names in this file are available in the *abcd_cbcls01_definitions.csv* file.

#### Bifactor scores.

The bifactor model was fit using confirmatory factor analyses as in Moore et al. (2020) and is described there in detail. Briefly, exploratory analyses were conducted on half of the baseline sample to determine which of the 119 CBCL items were most strongly associated with psychopathology. From these reduced sets, we extracted four interpretable factors and all CBCL items with a loading ≥ 0.40 on at least one factor were retained. Next, a confirmatory bifactor model in the second half of the sample was specified based on these results. As required for bifactor models, each retained CBCL item loaded on the general factor and only one specific factor. All other loadings were fixed to zero, and all factors were specified to be orthogonal. Factor models were fit in Mplus 8.3 using the mean- and variance-adjusted weighted least squares (WSLMV) estimator. All factor models accounted for the stratification of the sample in data collection sites, used poststratification weights, and accounted for clustering within families. Analyses made use of factor score estimates that were extracted from the confirmatory model (Clark et al., 2021).

#### HCP DSM-oriented scales.

Levels of psychopathology in the HCP sample were assessed via DSM-oriented scales (Barch et al., 2013) based on responses to the Achenbach Adult Self-Report (ASR) for ages 18–59 (Achenbach & Rescorla, 2007).

### Partial Least Squares

Partial least squares (PLS) analysis was used to find a latent variable that represents a Hurst-psychopathology pattern. PLS is a multivariate data analysis technique that decomposes the covariance matrix between two mean-centered datasets. Here, these datasets were the 1,839 by 392, subject by parcel matrix of *H* **X** and the 1,839 by 4, subject by factor matrix of extracted bifactor scores **Y**. Since these matrices are mean-centered, the covariance matrix **X**′**Y** can be decomposed via singular value decomposition so that

$$X^{\prime }Y=USV^{\prime }$$ (2)

where **U** is the 392 by 4 matrix of left singular vectors (brain loadings), **V** is the 4 by 4 matrix of right singular vectors (bifactor loadings or design loadings), and **S** is the 4 by 4 diagonal matrix of singular values (Krishnan et al., 2011) for the four fitted latent variables. The *i*th column of **U** and **V** represent the loadings of the *i*th latent variable. In the version of PLS used here, namely behavioral PLS, the design loadings are equivalent to the Pearson correlations between the extracted bifactor scores and the projection of the latent variable in U space or V space (Krishnan et al., 2011). The ratio of the squared *i*th singular value to the sum of all squared singular values gives the cross-block covariance explained by the *i*th latent variable and is used as a measure of effect size. The nonsignificant LVs explained 22%, 17%, and 11% of the covariance respectively while the significant LV explained 49% of the covariance.

Statistical significance of the PLS models was assessed by 10,000 permutations of the rows of the Hurst matrix **X** and comparing the observed cross-block covariance to permuted cross-block covariances. The stability of the left and right singular vectors (brain and bifactor loadings, respectively) is assessed by 10,000 bootstrap resamplings of both data matrices, **X** and **Y**. Bootstrap ratios are calculated as the empirical loading divided by the bootstrap variance and are distributed normally under the null (akin to a z-score). Thus, selecting an absolute threshold of 3 for stable loadings is equivalent to setting an alpha level of *p* < 0.001 for loadings different from zero.

### Pattern Score Computation

Pattern scores were computed in order to assess the degree to which participants expressed the latent relationship between *H* and psychopathology, which was found via PLS, that is, the degree to which participants exemplified the HPP. First, the PLS model was fit on ABCD data. Next, pattern scores were computed by correlating individual participants’ *H* maps with the HPP in order to assess spatial correspondence.

### Nonparticipation, Poststratification Weights, and Adjustment for Family Membership

Sensitivity analyses were conducted using poststratification and nonparticipation weights in an attempt to calibrate ABCD study sample distributions to nationally representative distributions as measured in the American Community Survey (ACS), and to correct for any biases due to not being included in the analysis relative to the demographic characteristics of the overall ABCD study sample, respectively. Procedures used by the ABCD consortium to calculate the poststratification scores (variable *abcd_acs_raked_propensity* in file *acspsw03.txt* of Curated Release 2.0.1) are described in detail elsewhere (Dick et al., 2021; Heeringa & Berglund, 2020). Briefly, a multiple logistic regression model was fit using concatenated ACS and ABCD data to predict study membership using participant variables age, sex, race/ethnicity, family income, family type, household size, parents’ workforce status, and Census Region. Weights were then raked to exact ACS population counts for age, sex, and race/ethnicity categories. Nonparticipation weights, calculated specifically for this study, were derived using an elastic net regularized binary logistic regression model using glmnet in R previously used in child psychopathology studies (Class et al., 2019). A binary variable indicating inclusion/exclusion in the analysis sample was the dependent variable, while age (in months), sex (male as reference category), race/ethnicity (non-Hispanic white as reference category), household size, years of maternal education, and square-root-transformed mean CBCL score were the independent variables. This elastic net model in glmnet was selected to derive the nonparticipation weights as it produces estimates with lower predictive errors than the full model, while accounting for redundant and highly correlated potential predictors (Hastie & Qian, 2014; Hastie, Tibshirani, & Friedman, 2009). The logistic regression model picked the optimal tuning parameter lambda with the least cross-validation deviance in model selection. Having selected the optimal model, probabilities $\hat{p}$ of response, conditional on being sampled, were calculated using the following equation:

$$\hat{p}=\text{exp}\left(X\hat{B}\right)/(1+\text{exp}\left(X\hat{B}\right)$$ (3)

where *X* is the model matrix and $\hat{B}$ is the vector of estimated parameters from the best model after cross-validation. Nonparticipation weights are thus the inverses of the probabilities $\hat{p}$. In order to compute corrected correlations between *H* in the HPP and future working memory performance, nonparticipation weights capturing which individuals had available 2-year follow-up data at the time of this study were also calculated. For the corrected correlations, poststratification weights, nonparticipation weights from the full ABCD study sample to this sample of 1,839 children, and nonparticipation weights from the baseline study sample to the future Release3.0 sample (*N* = 888) were multiplied together.

The nonparticipation and poststratification weights were multiplied prior to use in the PLS model. Next, the weighted mean, weighted variance, and weighted covariance were computed as (Becker, 2012)

$$\text{Weighted}\;\text{Mean}:\mu_{W}\left(X\right)=\frac{\sum w_{i}x_{i}}{\sum w_{i}}$$ (4)

$$\text{Weighted}\;\text{Variance}:\sigma_{W}^{2}\left(X\right)=\frac{\sum w_{i}{\left(x_{i}-\mu_{W}\right)}^{2}}{\sum w_{i}}$$ (5)

$$\text{Weighted}\;\text{Covariance}:cov_{W}\left(X,Y\right)=\frac{\sum w_{i}\left(x_{i}-\mu_{W}\left(X\right)\right)\left(y_{i}-\mu_{W}\left(Y\right)\right)}{\sum w_{i}}$$ (6)

with the resulting weighted covariance matrix then decomposed with singular value decomposition. The corrected correlation between *H* in the HPP and future working memory performance was calculated as:

$$r^{\text{corrected}}=\frac{cov_{W}\left(X,Y\right)}{\sqrt{\sigma_{W}^{2}\left(X\right)}\sqrt{\sigma_{W}^{2}\left(Y\right)}}$$ (7)

While the application of sampling weights in a behavioral PLS context is, to our understanding, novel, this method of calculating weighted means, variance, and covariance has been used elsewhere for closely related covariance decomposition techniques such as principle component analysis (Delchambre, 2015; Kelbert & Mozgunov, 2017), and canonical correlation analysis (Zhou, 2009) (which is very similar to PLS, i.e., PLS decomposes the covariance matrix with singular value decomposition and canonical correlation analysis decomposes the correlation matrix with singular value decomposition).

Statistical significance was assessed by computing the weighted mean and variance for the permuted brain matrix and nonpermuted bifactor matrix. Next, the weighted covariance matrix was calculated as

$$\text{Weighted}\;\text{Covariance}:\sigma_{W}\left(X,Y\right)=\frac{\sum \sqrt{w_{i}^{p}}\left(x_{i}-\mu_{W}\left(X\right)\right)\sqrt{w_{i}}\left(y_{i}-\mu_{W}\left(Y\right)\right)}{\sum w_{i}}$$ (8)

where $w_{i}^{p}$ is the weight obtained by permuting the weights alongside the rows of the brain matrix **X**. Finally, the resulting permuted covariance matrix was subject to SVD and the permuted cross-block covariance was compared to the observed, nonpermuted value.

First, we repeated the PLS analysis after randomly dropping all but one subject from each family, leaving 1,722 participants. This yielded a single statistically significant latent variable (*p* = .0017, covariance = 48%) with stable loadings on the general and ADHD factors (Supporting Information Figure S2A) and brain loadings significantly correlated with the unadjusted model (*r*_*s*_ = .98, Supporting Information Figure S3).

With these sampling weights (Garavan et al., 2018), PLS revealed a single statistically significant latent variable (*p* = .04, covariance = 38%) with stable loadings on the general, ADHD, and internalizing factors (Supporting Information Figure S2B) and brain loadings significantly correlated with the unadjusted model (*r*_*s*_ = .87, Supporting Information Figure S3).

Finally, we sought to confirm that the observed Hurst-psychopathology pattern is not specific to the bifactor model’s characterization of psychopathology. Thus, we repeated the PLS analysis with *t* scores derived from the 11 CBCL syndrome scale scores (Anxiety/Depression, Withdrawn/Depression, Somatic, Social, Thought, Attention, Rule Breaking, Aggressive, Internal, External, Total Problems; Achenbach & Ruffle, 2000), 6 CBCL DSM5 scale scores (Depression, Anxiety/Disordered, Somatic, ADHD, Oppositional, Conduct; Achenbach, Dumenci, & Rescorla, 2003), and 3 CBCL 2007 scale scores (Sluggish Cognitive Tempo, Obsessive-Compulsive, Stress; Achenbach & Rescorla, 2007). PLS revealed two statistically significant latent variables, the first of which (*p* = .017, covariance = 66%) had stable positive loadings on all derived CBCL scores (Supporting Information Figure S2C) and brain loadings significantly correlated with the unadjusted model (*r*_*s*_ = .84, Supporting Information Figure S3).

### Spatial Null Model

Spatial auto-correlation-preserving permutation tests were used to assess the statistical significance of correlations between Neurosynth terms and the Hurst-psychopathology pattern. These tests, termed “spin tests,” are necessary since standard permutation tests that assume no spatial auto-correlation significantly inflate false positive rates (Markello & Misic, 2021). We used the BrainSMASH python package (https://brainsmash.readthedocs.io/en/latest/) to generate parametric null brain maps with preserved spatial auto-correlation (SA) structure (Burt, Helmer, Shinn, Anticevic, & Murray, 2020). In short, BrainSMASH produces SA-preserving random maps whose variograms approximately match the variogram of an input brain map. Variograms are functions of spatial distance, d, which quantify the variance between all pairs of points that are a distance d away from each other. Here we use Euclidean distance calculated between the centroids of brain parcels.

### Functional Activation

Functional activation in the Destrieux 148 parcel cortical atlas (Destrieux, Fischl, Dale, & Halgren, 2010) were obtained from ABCD Release 2.0.1 tabulated imaging data available on the NIMH data archive (https://nda.nih.gov/abcd). After preprocessing, beta weights for a linear contrast between 2-back and 0-back blocks were computed by a generalized linear model with motion estimates, derivatives, squared estimates, and squared derivatives included as nuisance regressors (Hagler et al., 2019). The hemodynamic response function was modeled as gamma functions with temporal derivatives and convolved with square waves indicating each block. Average beta coefficients across runs of the 2-back versus 0-back contrast were used to assess functional activation. These data were retrieved from the abcd_tfncr1bwdp201.txt and abcd_tfncr1bwdp201.txt files.

To compare the correlation between activations and pattern scores to the Hurst-psychopathology pattern, the activations were resampled via averaging into the 392 parcels of the Craddock atlas with the nilearn (Abraham et al., 2014) function NiftiLabelsMasker.

### *k*-Fold Cross-Validation

In order to assess the generalizability of the PLS results we employed a k-Fold cross-validation approach (Dinga et al., 2019; Marek et al., 2022) to determine the consistency of the HPP in different subsets of the full sample and to determine the out-of-sample performance of relationships between HPP and behavioral measures of working memory performance and psychopathology. We employed stratified k-fold sampling (using the sklearn StratifiedKFold class in Python) to create folds (*k* ∈ [2, 40]) that were matched on age, CBCL score, birth sex, and race/ethnicity.

For each of the k folds we re-ran the PLS analysis leaving out the $\frac{1}{k}$ fraction of the data in the given fold. In order to assess the consistency of the HPP, we correlated the brain loadings found for each fold with the HPP result found using all of the data and benchmarked those correlations against spin nulls (Markello & Misic, 2021). In order to assess relationships between the latent variable and behavior, we computed out-of-sample brain scores by taking the dot product between the brain loadings and the left-out *H* maps. These out-of-sample brain scores were then correlated with 2-back accuracy and extracted factor scores for the general factor of psychopathology.

In order to determine sample size appropriate confidence intervals across the different fold sizes, we first computed the appropriate z-score threshold as

$$Z_{\mathrm{threshold}}=PPF\left(1-\frac{\alpha }{2}\right)\frac{1}{\sqrt{{\mathrm{length}}_{k}-3}}$$

where PPF is the percentage point function for the standard normal distribution, *α* is the significance threshold set at 0.05, and *length*_*k*_ is the number of samples in each fold for a given *k*. Next we computed the Fisher r-to-Z transformation for the correlations, *r*, as

$$Z_{r}=\text{log}\left[\frac{1+r}{1-r}\right]\frac{1}{2}$$

finally, we computed the confidence interval as

$$CI_{r}=r\pm \frac{e^{2\left(Z_{r}-Z_{\text{threshold}}\right)}-1}{e^{2\left(Z_{r}-Z_{\text{threshold}}\right)}+1}$$

### Software

ABCD neuroimaging data were preprocessed with a custom version of fMRIPREP available at https://github.com/enlberman/uchicagoABCDProcessing. PLS models were run in MATLAB via a modified version (available at https://github.com/enlberman/WeightedPLS) of the Rotman Baycrest PLS software developed specifically for neuroimaging data (https://www.rotman-baycrest.on.ca/index.php?section=84). Nonparticipation weights were computed in R using the “glmnet” and “foreign” packages. All other analyses were performed and figures created in python with standard packages (code available at https://github.com/enlberman/abcd_enback_bifactor).

## ACKNOWLEDGMENTS

Data used in the preparation of this article were obtained from the Adolescent Brain Cognitive DevelopmentSM (ABCD) Study (https://abcdstudy.org), held in the NIMH Data Archive (NDA). This is a multisite, longitudinal study designed to recruit more than 10,000 children age 9–10 and follow them over 10 years into early adulthood. The ABCD Study is supported by the National Institutes of Health and additional federal partners under award numbers U01DA041048, U01DA050989, U01DA051016, U01DA041022, U01DA051018, U01DA051037, U01DA050987, U01DA041174, U01DA041106, U01DA041117, U01DA041028, U01DA041134, U01DA050988, U01DA051039, U01DA041156, U01DA041025, U01DA041120, U01DA051038, U01DA041148, U01DA041093, U01DA041089, U24DA041123, U24DA041147. A full list of supporters is available at https://abcdstudy.org/federal-partners.html. A listing of participating sites and a complete listing of the study investigators can be found at https://abcdstudy.org/consortium_members/. ABCD consortium investigators designed and implemented the study and/or provided data but did not necessarily participate in the analysis or writing of this report. This manuscript reflects the views of the authors and may not reflect the opinions or views of the NIH or ABCD consortium investigators.

The ABCD data repository grows and changes over time. The ABCD data used in this report came from DOI 10.15154/1503209 and DOI 10.15154/1519007. DOIs can be found at https://dx.doi.org/10.15154/1503209 and https://dx.doi.org/10.15154/1519007.

Data were provided (in part) by the Human Connectome Project, WU-Minn Consortium (Principal Investigators: David Van Essen and Kamil Ugurbil; 1U54MH091657) funded by the 16 NIH Institutes and Centers that support the NIH Blueprint for Neuroscience Research; and by the McDonnell Center for Systems Neuroscience at Washington University.

This research also benefited from the ABCD Workshops on Brain Development, Mental Health, and Longitudinal Modeling, supported by the NIMH and NIH under award numbers R25MH120869, R25MH125545, and UG3DA045251. This work was partially supported by S&CC-1952050 (to M.G.B.), R00MH117274 (to A.N.K.), and UG3DA045251 (to B.B.L.). The use of the ABCD and HCP datasets was approved by the University of Chicago Institutional under IRB18-0476 and IRB21-1045 respectively. Access to the data was restricted by password and two-factor authentication to individuals approved by the IRB and the Data Use Agreements.

## SUPPORTING INFORMATION

Supporting information for this article is available at https://doi.org/10.1162/netn_a_00322. ABCD data are publicly available and access can be requested at https://abcdstudy.org/. HCP data are publicly available and access can be requested at https://db.humanconnectome.org/. All relevant code is available on github: https://github.com/enlberman/uchicagoABCDProcessing and https://github.com/enlberman/abcd_enback_bifactor. The corresponding authors A. J. Stier and M. G. Berman can be contacted at andrewstier@uchicago.edu and bermanm@uchicago.edu for further questions.

## AUTHOR CONTRIBUTIONS

Andrew Jacob Stier: Conceptualization; Formal analysis; Investigation; Methodology; Software; Visualization; Writing – original draft; Writing – review & editing. Carlos Cardenas-Iniguez: Formal analysis; Investigation; Methodology; Writing – review & editing. Omid Kardan: Formal analysis; Investigation; Methodology; Writing – review & editing. Tyler M. Moore: Formal analysis; Writing – review & editing. Francisco A. C. Meyer: Writing – review & editing. Monica D. Rosenberg: Funding acquisition; Supervision; Writing – review & editing. Antonia N. Kaczkurkin: Funding acquisition; Writing – review & editing. Benjamin B. Lahey: Formal analysis; Funding acquisition; Supervision; Writing – review & editing. Marc G. Berman: Conceptualization; Funding acquisition; Methodology; Supervision; Writing – review & editing.

## FUNDING INFORMATION

This research benefited from the ABCD Workshops on Brain Development, Mental Health, and Longitudinal Modeling, supported by the NIMH and NIH under award numbers R25MH120869, R25MH125545, and UG3DA045251. This work was partially supported by S&CC-1952050 (to M.G.B.), R00MH117274 (to A.N.K.), and UG3DA045251 (to B.B.L.).

## TECHNICAL TERMS

Psychopathology: An overarching term for mental disorders.
Cognitive resources: This refers to the information processing capabilities of the brain which may be allocated to various cognitive processes.
Hurst exponent: A measure of scale-free dynamics for time series.
Scale-free: When brain signals have a 1/*f*-like power spectrum and look similar when zoom-in or zoomed-out they are scale-free.
Working memory: The cognitive processes that allow for temporary holding of information for processing and manipulation.
Critical state: A system state that is at or near a transition between different functional regimes.
Cognitive impairment: Any reduction in cognitive performance from expectation or baseline.
PLS: Partial least squares is a multivariate data analysis technique often used to associated brain activity and behavior.
Fronto-parietal: Frontal and parietal brain areas that are often associated with higher order cognitive processes.
Sensorimotor: Brain areas that are often associated with processing sensory information and directing movements.
