# Supplementary material for: A pattern of cognitive resource disruptions in childhood psychopathology
Source: Netw Neurosci. 2023 Oct 1;7(3):1153–80. doi: 10.1162/netn_a_00322 (PMC10473262; doi:10.1162/netn_a_00322)
Supplement: Supplementary file 1 [file netn-7-3-1153-s001.pdf]

Supplementary materials

Supplementary Text

Neurosynth Term Sets

For each of the two discovered term sets we briefly reviewed the literature associated with each term and discuss how the term contributes conceptually to its broader category of task-cognition or cue-response.

Task-Cognition

These terms all cover various cognitive constructions that are expected to be engaged during the emotional n-back task analyzed in this study which simultaneously probes emotional social information processing and working memory performance.

"Memory Retrieval": This term has 228 associated studies in the Neurosynth database. As an example, the top five of these studies with the largest loadings on this term operationalize "memory retrieval" by comparing individuals with amnestic mild cognitive impairment against controls (*1*), comparing auto-biographical and laboratory-based memory tasks (*2*), free recall of word lists (*3, 4*), and a thematic apperception test that invokes memories of aversive interpersonal relationships (*5*).

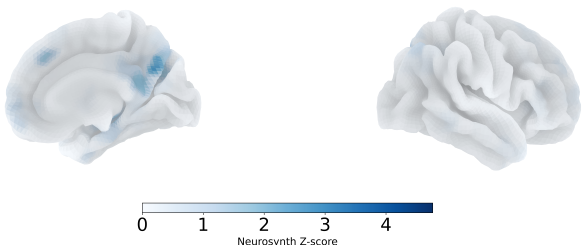

Figure 1: The term map for "Memory Retrieval" from Neurosynth.

"Social Cognition": This term has 220 associated studies in the Neurosynth database. As an example, the top five of these studies with the largest loadings on this term operationalize "social cognition" by judging the trustworthiness of faces (*6*), self-report scales on social responsive-

ness and empathy (*7*), a theory of mind task (*8*), encoding facts about unfamiliar people (*9*), and 10 weeks of social cognitive training (*10*).

"Autobiographical Memory": This term has 84 associated studies in the Neurosynth database. As an example, the top five of these studies with the largest loadings on this term operationalize "autobiographical memory" by recalling a narrative account about a distressing event (*11*), by recalling specific autobiographical memory details over 3 years in dementia patients (*12*), comparing individuals with transient epileptic amnesia and controls (*13*), contrasting recent and remote autobiographical memories (*14*), and contrasting autobiographical memories of different emotional valence (*15*).

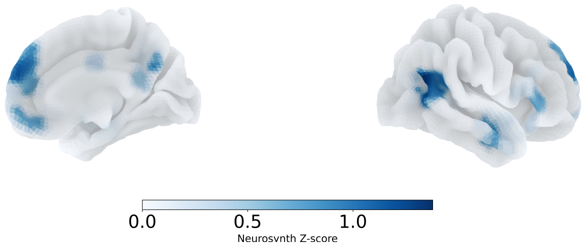

Figure 2: The term map for "Social Cognition" from Neurosynth.

"Semantic Memory": This term has 123 associated studies in the Neurosynth database. As an example, the top five of these studies with the largest loadings on this term operationalize "semantic memory" by a verbal object recall task (*16*), a task requiring the recall of objects from different categories (*17*), a famous name discrimination task (*18*), a task requiring the recall of well known public events (*19*), and a task requiring the recall of common facts or the location of famous landmarks (*20*).

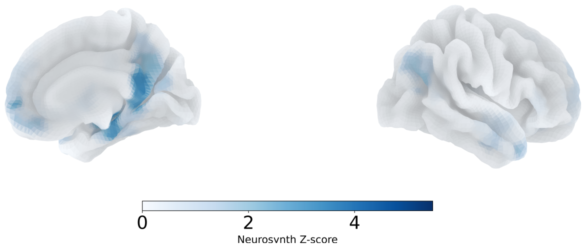

Figure 3: The term map for "Autobiographical Memory" from Neurosynth.

"Recall": This term has 244 associated studies in the Neurosynth database. As an example, the top five of these studies with the largest loadings on this term operationalize "recall" by retrieval of previously studies items (*21*), famous name recall (*22*), free recall (*23*), forward and backward digit recall (*24*), and recognition of previously encoded pictures (*25*).

"Knowledge": This term has 635 associated studies in the Neurosynth database. As an example, the top five of these studies with the largest loadings on this term operationalize "knowledge" by asking participants to pair objects with similar manipulation properties or use contexts (*26*), asking participants to find new scientific problems (*27*), testing schematic knowledge in medical school students (*28*), examining semantic decisions that rely on sensory knowledge (*29*), and by assessing object color verification and color attribute judgement (*30*).

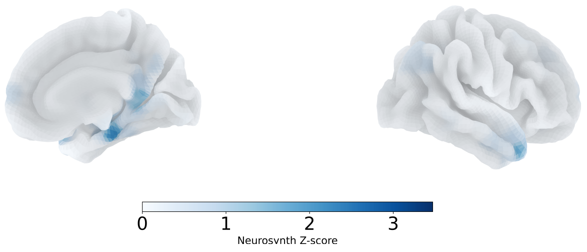

Figure 4: The term map for "Semantic Memory" from Neurosynth.

Cue-Response

These terms cover cognitive constructs that are expected to be involved in the aspects of the EN-back task which require attending to the task cues and executing responses. For example, areas related to "coordination", "movement", "rhythm", and "motor control" are expected to be involved in coordinating responses between the left hand and the right hand and pressing the proper response button. In addition, areas related to "speech production", and "rehearsal", are likely to be, at least partially engaged during the attending to and remembering of the directions of the task. Similarly, we would expect areas related to "spatial attention" and "effort" to be engaged while attending to stimuli throughout the task.

“Planning”: This term has 258 associated studies in the Neurosynth database. As an example, the top five of these studies with the largest loadings on this term operationalize “planning” by asking participants to plan solutions to tower of hanoi problems (*31, 32*), planning four digit finger sequences (*33*), planning of tool and communication-related gestures (*34*), and while performing tower of London tasks (*35*).

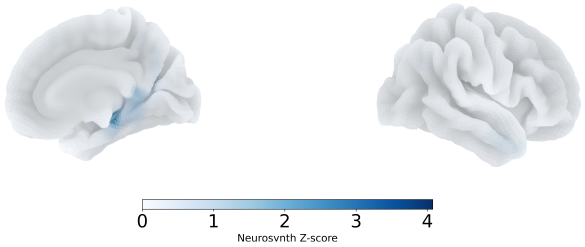

Figure 5: The term map for “Recall” from Neurosynth.

“Coordination”: This term has 150 associated studies in the Neurosynth database. As an example, the top five of these studies with the largest loadings on this term operationalize “coordination” by coordinated hand and foot movement patterns (*36*), conversational exchanges (*37*), a visuomotor coordination task (*38*), bimanual movement tasks (*39*), and complex rhythmic tapping of both hands (*40*).

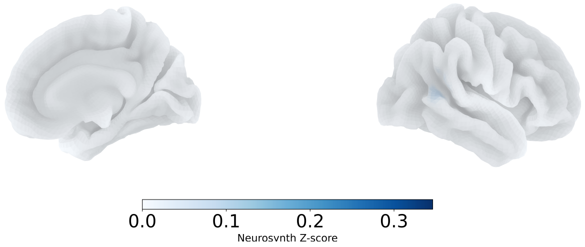

Figure 6: The term map for “Knowledge” from Neurosynth.

“Movement”: This term has 670 associated studies in the Neurosynth database. As an example, the top five of these studies with the largest loadings on this term operationalize “movement” by comparing individuals with hand paralysis with controls (*41*), performance of a motor task at different movement rates (*42*), examining the switch from rest to a right-hand movement task (*43*), investigating involuntary muscle contraction (*44*), and observing imagined, observed, and passive wrist movements (*45*).

"Rhythm": This term has 76 associated studies in the Neurosynth database. As an example, the top five of these studies with the largest loadings on this term operationalize "rhythm" by an auditory rhythm discrimination task (*46*), having participants memorize and reproduce auditory and visual rhythmic information (*47, 48*), having participants memorize various rhythms (*49*), and listening to various rhythms (*50*).

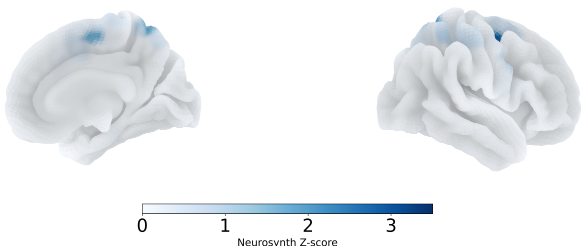

Figure 7: The term map for "Planning" from Neurosynth.

"Speech Production": This term has 107 associated studies in the Neurosynth database. As an example, the top five of these studies with the largest loadings on this term operationalize "" by studies of reading aloud (*51*), overt speech production (*52*), having participants produce simple and complex syllable sequences (*53*), having participants plan speech (*54*), and the performance of language and speech (*55*).

"Spatial Attention": This term has 149 associated studies in the Neurosynth database. As an example, the top five of these studies with the largest loadings on this term operationalize "spatial attention" by a cued auditory spatial attention task (*56, 57*), separate focused and distributed visuospatial attention tasks (*58*), a cued spatial attention task (*59*), and lateralized spatial attention tasks (*60*).

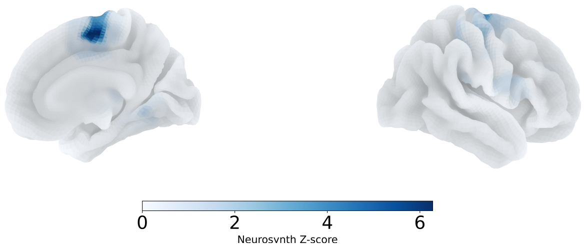

Figure 8: The term map for "Coordination" from Neurosynth.

"Rehearsal": This term has 74 associated studies in the Neurosynth database.

As an example, the top five of these studies with the largest loadings on this term operationalize "rehearsal" by a spatial working memory task (*61*), a verbal-short-term memory task (*62*), mental rehearsal of a golf swing (*63*), recordings of autobiographical memory-related audio (*64*), and a vocabulary learning task (*65*).

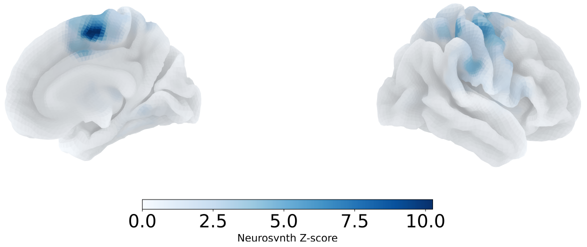

Figure 9: The term map for "Movement" from Neurosynth.

"Effort": This term has 204 associated studies in the Neurosynth database. As an example, the top five of these studies with the largest loadings on this term operationalize "effort" by asking participants to choose between a harder and easier cognitive task (*66*), having participants choose between easier and harder motor tasks (*67*), having participants discount the value of delayed and effortful rewards (*68*), swallowing liquids when thirsty and overdrunk (*69*), and emotional stimulation before physical exertion (*70*).

"Motor Control": This term has 214 associated studies in the Neurosynth database. As an example, the top five of these studies with the largest loadings on this term operationalize "motor control" by performing a motor control task while experiencing pain (*71*), a task combining emotional and sensorimotor stimuli (*72*), making participants squeeze a soft material (*73*), observing manual pointing movements (*74*), and n (*75*).

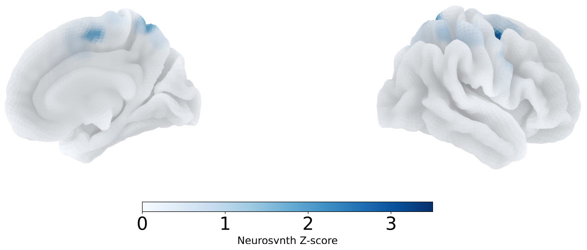

Figure 10: The term map for "Planning" from Neurosynth.

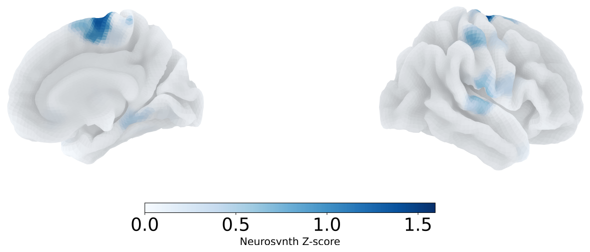

Figure 11: The term map for “Rhythm” from Neurosynth.

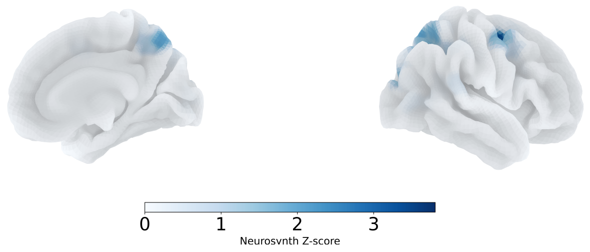

Figure 12: The term map for “Spatial Attention” from Neurosynth.

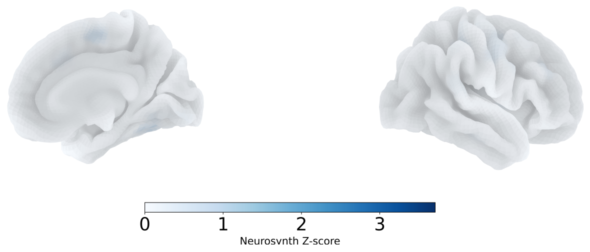

Figure 13: The term map for “Rehersal” from Neurosynth.

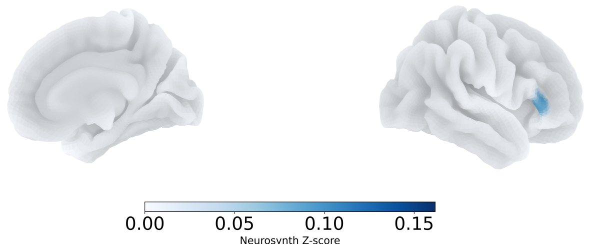

Figure 14: The term map for “Effort” from Neurosynth.

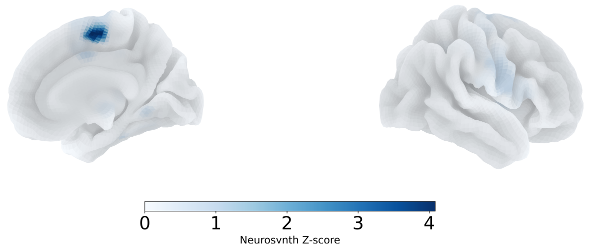

Figure 15: The term map for “Motor Control” from Neurosynth.

References

1. F. Bai, Y. Yuan, H. Yu, Z. Zhang, *Behavioural brain research* **308**, 38 (2016).
2. H. Kim, *Neuroimage* **61**, 966 (2012).
3. C. Mensebach, *et al.*, *Psychiatry Research: Neuroimaging* **171**, 94 (2009).
4. J. S. Phillips, K. Velanova, D. A. Wolk, M. E. Wheeler, *Neuroimage* **46**, 1209 (2009).
5. K. Schnell, T. Dietrich, R. Schnitker, J. Daumann, S. Herpertz, *Journal of affective disorders* **97**, 253 (2007).
6. D. Baas, *et al.*, *Neuroimage* **40**, 719 (2008).
7. S. M. Lazar, D. W. Evans, S. M. Myers, A. Moreno-De Luca, G. J. Moore, *Behavioural brain research* **263**, 1 (2014).
8. C. J. McAdams, D. C. Krawczyk, *Psychiatry Research: Neuroimaging* **194**, 54 (2011).
9. W. K. Simmons, M. Reddish, P. S. Bellgowan, A. Martin, *Cerebral Cortex* **20**, 813 (2010).
10. C. I. Hooker, *et al.*, *Schizophrenia research* **139**, 53 (2012).
11. M. G. Whalley, M. D. Rugg, C. R. Brewin, *Psychiatry Research: Neuroimaging* **201**, 98 (2012).
12. E. A. Maguire, D. Kumaran, D. Hassabis, M. D. Kopelman, *Neuropsychologia* **48**, 123 (2010).
13. F. Milton, C. R. Butler, A. Benattayallah, A. Z. Zeman, *Neuropsychologia* **50**, 3528 (2012).
14. S. Steinvorth, S. Corkin, E. Halgren, *Neuroimage* **30**, 285 (2006).

15. M. Piefke, P. H. Weiss, H. J. Markowitsch, G. R. Fink, *Human brain mapping* **24**, 313 (2005).

16. M. Assaf, *et al.*, *Biological psychiatry* **59**, 452 (2006).

17. M. Grossman, *et al.*, *Brain* **126**, 292 (2003).

18. J. C. Smith, *et al.*, *Neuroimage* **54**, 635 (2011).

19. S. Oddo, *et al.*, *Cortex* **46**, 29 (2010).

20. S. M. Hoscheidt, L. Nadel, J. Payne, L. Ryan, *Behavioural brain research* **212**, 121 (2010).

21. A. Maril, J. S. Simons, J. P. Mitchell, B. L. Schwartz, D. L. Schacter, *Neuroimage* **18**, 827 (2003).

22. S. Yagishita, *et al.*, *Neuroimage* **41**, 1142 (2008).

23. N. M. Long, I. Öztekin, D. Badre, *Journal of Neuroscience* **30**, 10967 (2010).

24. X. Sun, *et al.*, *Neuroimage* **26**, 36 (2005).

25. G. Pergola, A. Ranft, K. Mathias, B. Suchan, *Neuroimage* **74**, 195 (2013).

26. N. Canessa, *et al.*, *Cerebral Cortex* **18**, 740 (2008).

27. T. Dandan, *et al.*, *Experimental brain research* **228**, 437 (2013).

28. G. Brod, U. Lindenberger, A. D. Wagner, Y. L. Shing, *Journal of Neuroscience* **36**, 8103 (2016).

29. R. F. Goldberg, C. A. Perfetti, W. Schneider, *Journal of Neuroscience* **26**, 4917 (2006).

30. X. Wang, *et al.*, *Neuroimage* **76**, 252 (2013).

31. G. Wagner, K. Koch, J. R. Reichenbach, H. Sauer, R. G. Schlösser, *Neuropsychologia* **44**, 2337 (2006).

32. C. Crescentini, S. Seyed-Allaei, A. Vallesi, T. Shallice, *Neuropsychologia* **50**, 1521 (2012).

33. J. Jankowski, L. Scheef, C. Hüppe, H. Boecker, *Neuroimage* **44**, 1369 (2009).

34. S. Bohlhalter, *et al.*, *Cerebral cortex* **19**, 1256 (2009).

35. J. P. Trujillo, *et al.*, *Human Brain Mapping* **36**, 3703 (2015).

36. S. P. Swinnen, *et al.*, *Neuroimage* **49**, 2570 (2010).

37. M. L. Healey, *et al.*, *Neuropsychologia* **69**, 56 (2015).

38. S. Koeneke, K. Lutz, T. Wüstenberg, L. Jäncke, *Neuroimage* **22**, 1336 (2004).

39. N. Wenderoth, F. Debaere, S. Sunaert, S. P. Swinnen, *European Journal of Neuroscience* **22**, 235 (2005).

40. F. Ullén, H. Forssberg, H. H. Ehrsson, *Journal of Neurophysiology* **89**, 1126 (2003).

41. M. Burgmer, *et al.*, *Neuroimage* **29**, 1336 (2006).

42. Y. Tanaka, *et al.*, *Experimental brain research* **193**, 143 (2009).

43. S. Treserras, *et al.*, *Neuroimage* **48**, 207 (2009).

44. A. Parkinson, M. McDonagh, R. Vidyasagar, *Brain research* **1304**, 57 (2009).

45. A. J. Szameitat, S. Shen, A. Conforto, A. Sterr, *Neuroimage* **62**, 266 (2012).

46. S.-E. Chang, H. M. Chow, E. A. Wieland, J. D. McAuley, *NeuroImage: Clinical* **12**, 442 (2016).

47. N. Konoike, *et al.*, *Neuroimage* **63**, 328 (2012).

48. N. Konoike, *et al.*, *PloS one* **10**, e0130120 (2015).

49. K. Sakai, *et al.*, *Journal of Neuroscience* **19**, 10074 (1999).

50. S. L. Bengtsson, *et al.*, *cortex* **45**, 62 (2009).

51. C. J. Price, *et al.*, *Neuroimage* **29**, 643 (2006).

52. S. Brown, *et al.*, *Brain and cognition* **70**, 31 (2009).

53. P. Tremblay, I. Deschamps, *Brain Structure and Function* **221**, 3275 (2016).

54. C. A. Kell, B. Morillon, F. Kouneiher, A.-L. Giraud, *Cerebral Cortex* **21**, 932 (2011).

55. A. Oh, E. G. Duerden, E. W. Pang, *Brain and language* **135**, 96 (2014).

56. C.-T. Wu, D. H. Weissman, K. C. Roberts, M. G. Woldorff, *Brain research* **1134**, 187 (2007).

57. L. Kong, *et al.*, *Cerebral Cortex* **24**, 773 (2014).

58. W. Sturm, *et al.*, *Experimental Brain Research* **171**, 16 (2006).

59. J. J. Geng, G. R. Mangun, *Journal of cognitive neuroscience* **21**, 1584 (2009).

60. D. R. Gitelman, *et al.*, *Brain* **122**, 1093 (1999).

61. B. Postle, E. Awh, J. Jonides, E. Smith, M. D'Esposito, *Cognitive Brain Research* **20**, 194 (2004).

62. R. Henson, N. Burgess, C. D. Frith, *Neuropsychologia* **38**, 426 (2000).

63. L. Bezzola, S. Mérillat, L. Jäncke, *Frontiers in human neuroscience* **6**, 67 (2012).

64. E. Svoboda, B. Levine, *Journal of neuroscience* **29**, 3073 (2009).

65. D. E. Callan, N. Schweighofer, *Human brain mapping* **31**, 645 (2010).

66. N. Schouppe, J. Demanet, C. N. Boehler, K. R. Ridderinkhof, W. Notebaert, *Journal of Neuroscience* **34**, 2148 (2014).

67. I. T. Kurniawan, *et al.*, *Journal of neurophysiology* **104**, 313 (2010).

68. S. A. Massar, C. Libedinsky, C. Weiyan, S. A. Huettel, M. W. Chee, *Neuroimage* **120**, 104 (2015).

69. P. Saker, M. J. Farrell, G. F. Egan, M. J. McKinley, D. A. Denton, *Proceedings of the National Academy of Sciences* **113**, 12274 (2016).

70. L. Schmidt, *et al.*, *Journal of Neuroscience* **29**, 9450 (2009).

71. G. Misra, S. A. Coombes, *Cerebral cortex* **25**, 1906 (2015).

72. T. Hassa, *et al.*, *NeuroImage: Clinical* **15**, 143 (2017).

73. F. Cui, *et al.*, *PLoS One* **9**, e84367 (2014).

74. B. Lorey, *et al.*, *Neuroimage* **49**, 3239 (2010).

75. M. Vink, *et al.*, *Human brain mapping* **25**, 336 (2005).

Supplementary Figures

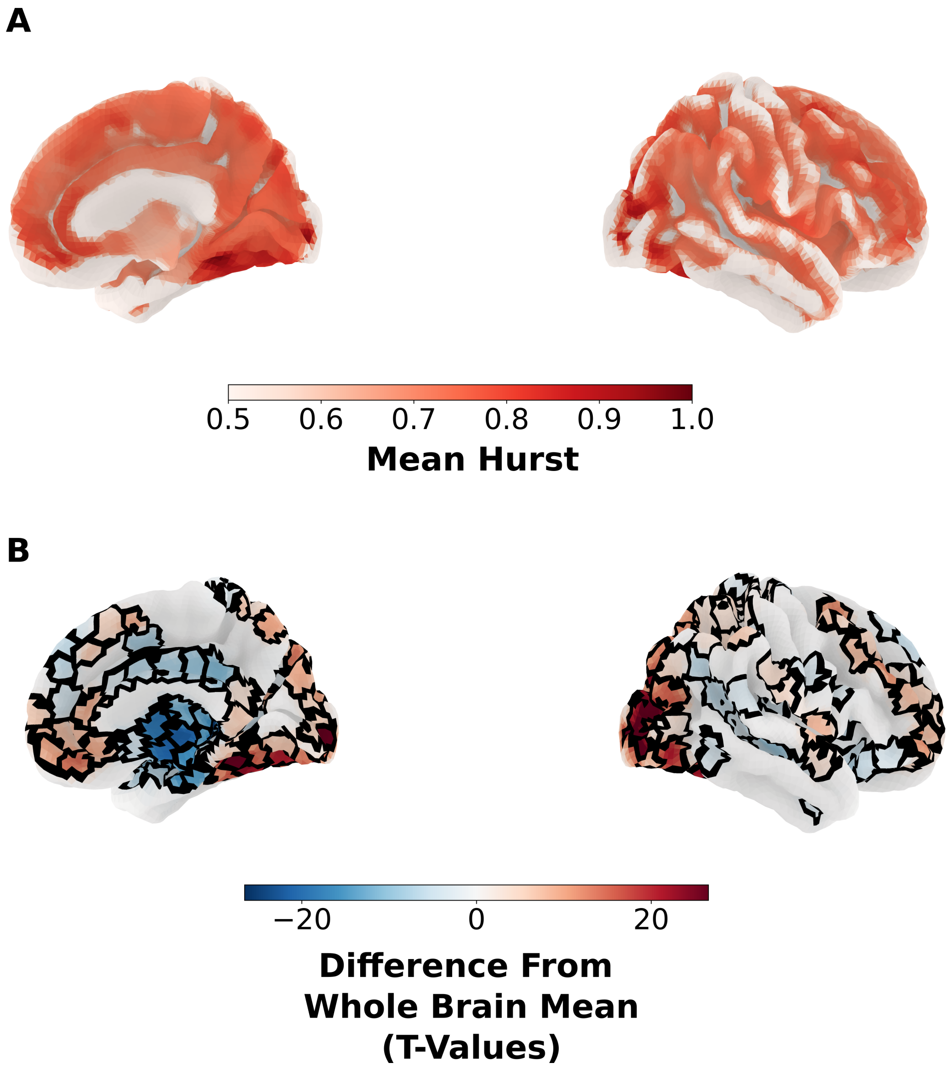

Supplementary Figure 1: A) Average Hurst values in the ABCD dataset. B) Significant t-values (outlined) for differences from average whole brain Hurst values after Benjimini-Hochberg correction for multiple comparisons at $\alpha = .05$

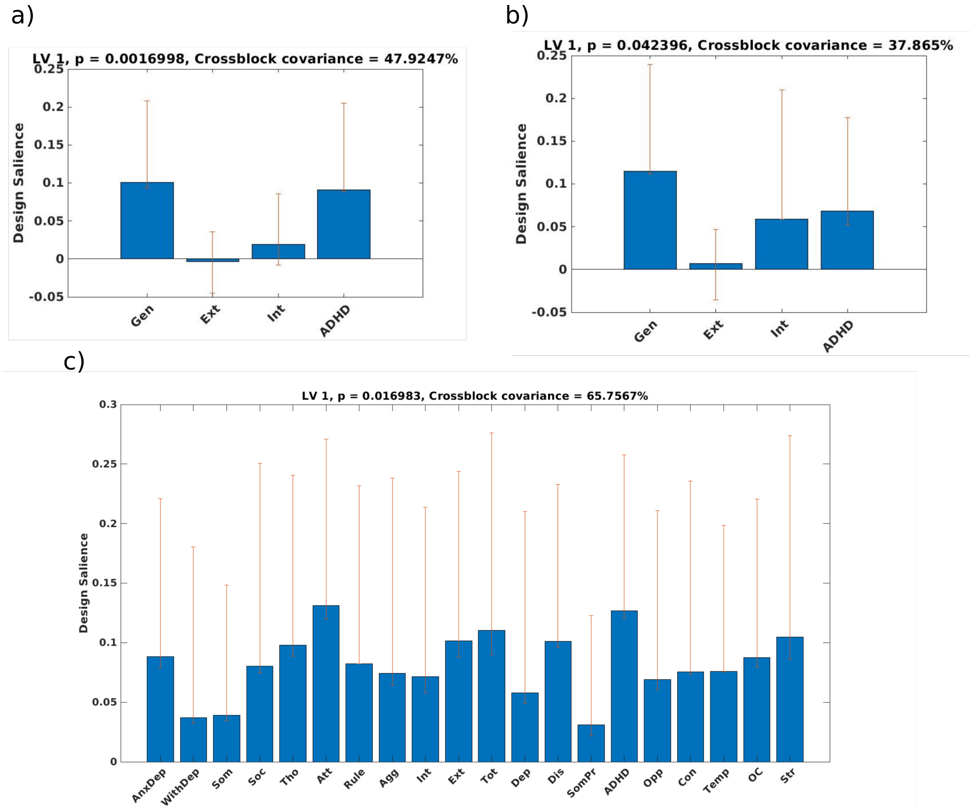

Supplementary Figure 2: Design loadings for PLS sensitivity models. These plots show the behavioral loadings on the significant latent variable for each of the sensitivity tests. a) Only one member of each family was retained. b) Non-participation and post-stratification weights were applied. c) CBCL scales were used instead of extracted bifactor scores. AnxDep = Anxiety Depression Syndrome Scale; WithDep = Withdrawn Depression Syndrome Scale; Som = Somatic Syndrome Scale; Soc = Social Syndrome Scale; Tho = Thought Syndrome Scale; Att = Attention Syndrome Scale; Rule = Rule Breaking Syndrome Scale; Agg = Aggressive Syndrome Scale; Int = Internal Syndrome Scale; Ext = External Syndrome Scale; Tot = Total Problems Syndrome Scale; Dep = Depression DSM5 Scale; Dis = Anxiety/Disordered DSM5 Scale; SomPr = Somatic Problems DSM5 Scale; ADHD = ADHD DSM5 Scale; Opp = Oppositional DSM5 Scale; Con = Conduct DSM5 Scale; Temp = Sluggish Cognitive Tempo 2007 Scale; OC = Obsessive-Compulsive 2007 Scale; Str = Stress 2007 Scale.

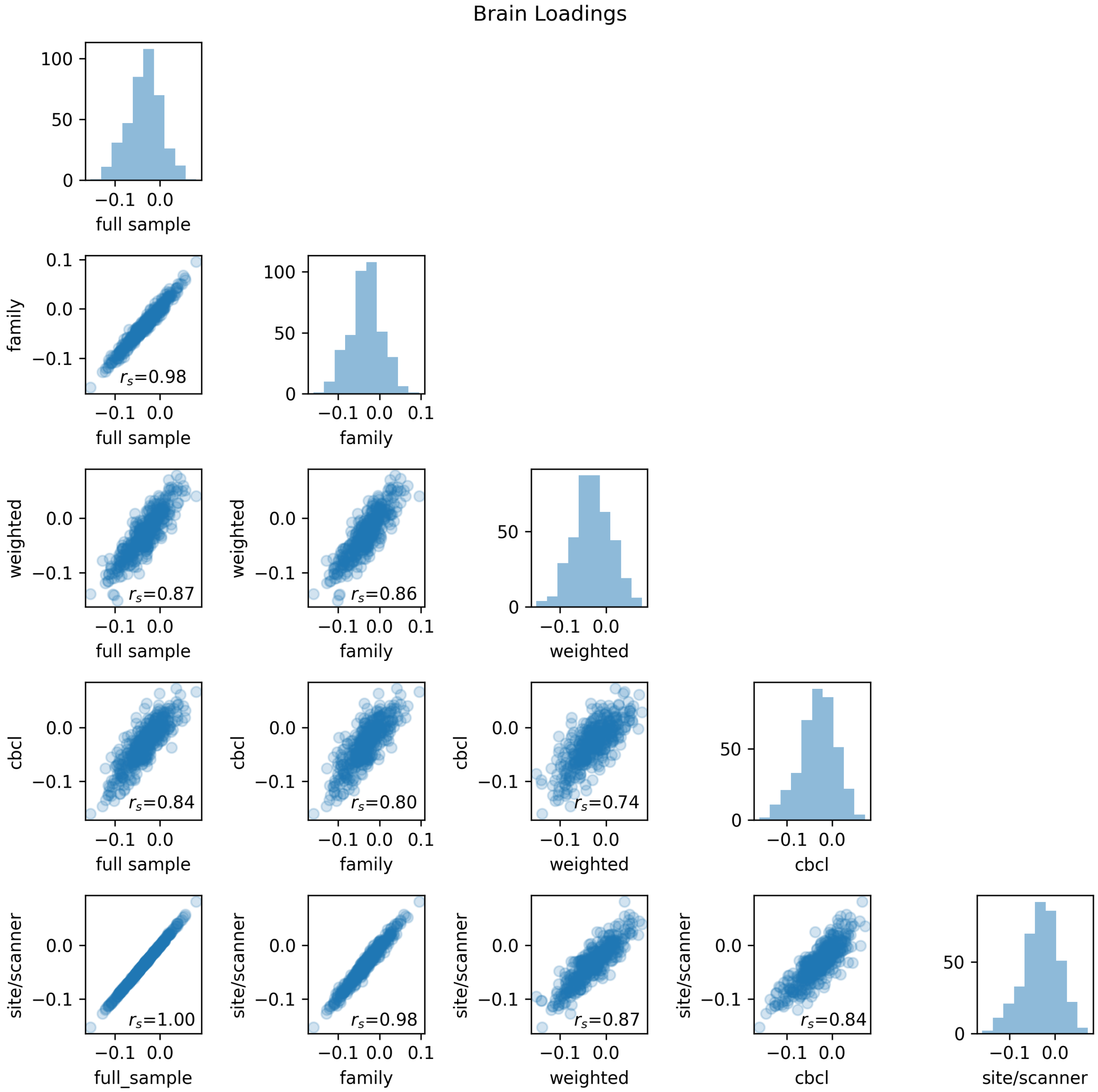

Supplementary Figure 3: Comparison of brain loadings between the un-adjusted full sample model, the model where only one family member was retained, the model adjusted for post-stratification and non-participation, the model with CBCL scores instead of extracted bifactor scores, and the model where data was residualized with respect to site and scanner. All correlations are significant.

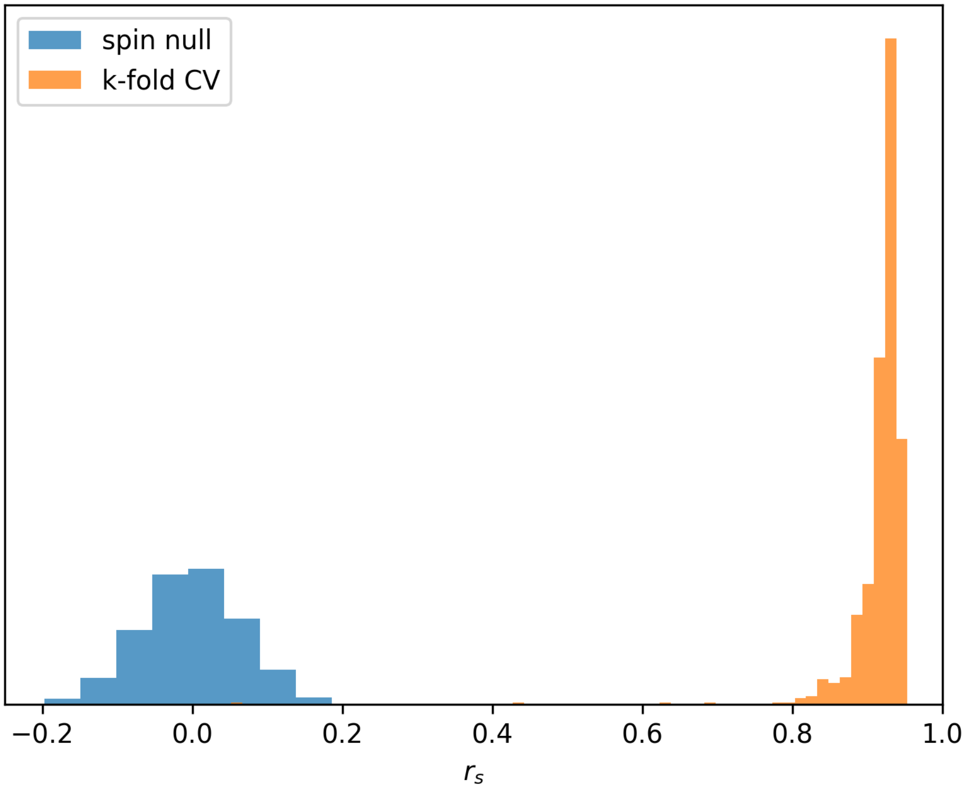

Supplementary Figure 4: The HPP (brain loadings) from each fold of k-fold cross validation are highly correlated with the HPP for the full sample and are significantly different from the spin null model of the full sample HPP which preserves spatial auto-correlation.

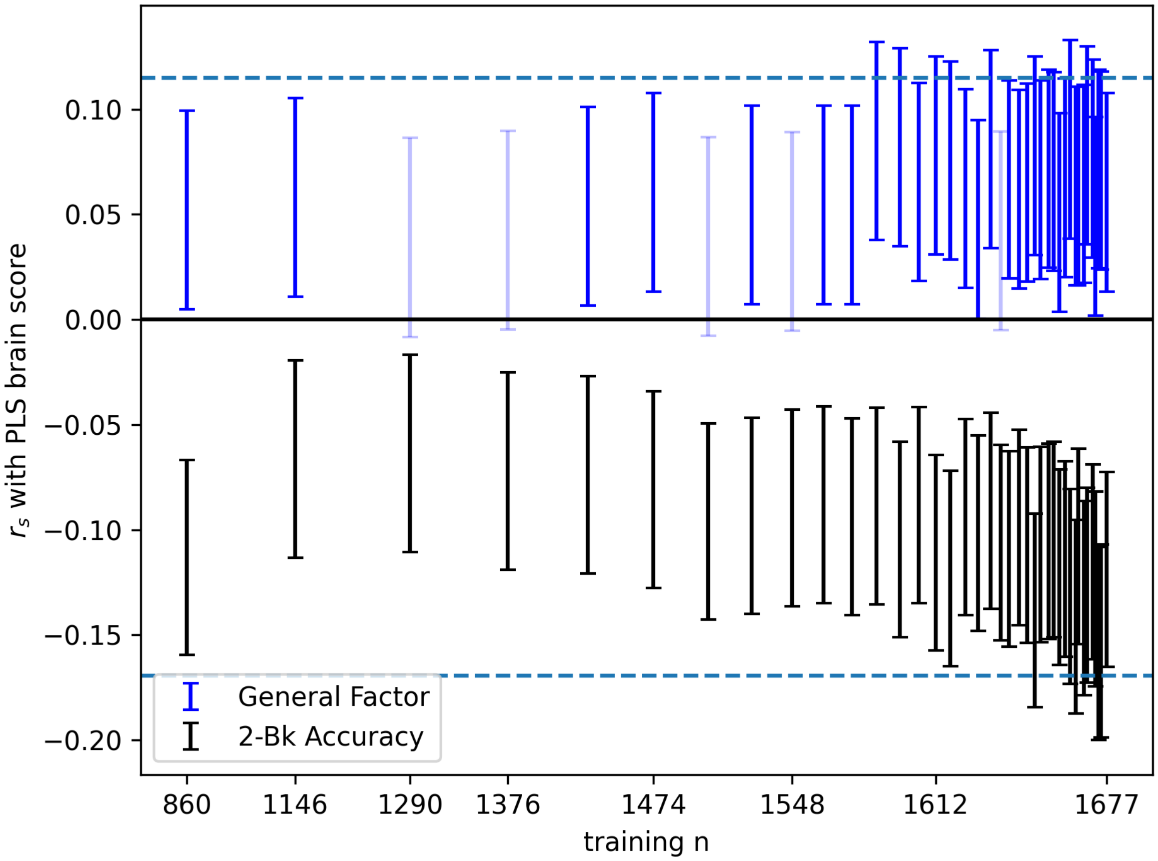

Supplementary Figure 5: The relationship between the HPP and behavior replicates out of sample in k-fold cross validation. Correlations between the PLS brain scores, i.e., HPP pattern scores, and behavior are significant across most values of k. The x-axis shows the training n for each fold which increases as the number of folds goes from k=2 to k=40. Correlations between the HPP and behavior from the model fit using the entire dataset are shown by the dashed blue line.

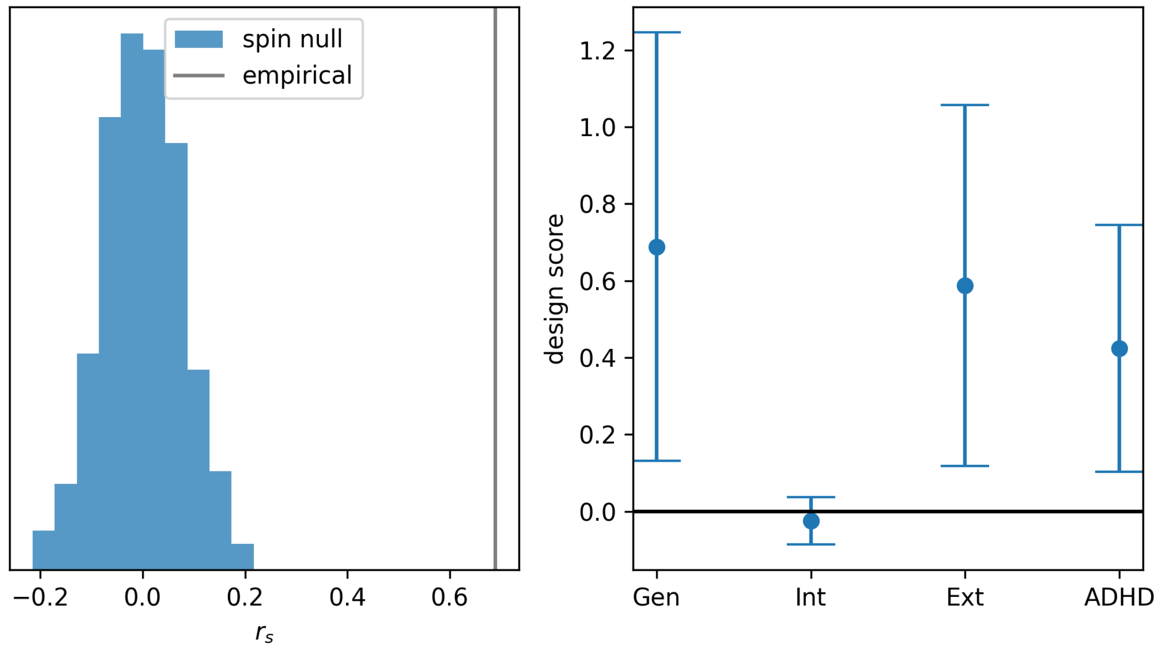

Supplementary Figure 6: Motion censoring sensitivity test. We re-ran the PLS analysis with H calculated from BOLD time series where frames with framewise displacement $>$ .2mm were censored. This has been shown to reduce respiratory-artifact-related brain-behavior false positives. Left) Brain loadings, i.e., the HPP is highly similar whether or not high-motion frames are censored. The correlation is significant relative to spin nulls that account for spatial autocorrelation. Right) The design scores are highly similar whether or not high-motion frames are censored.

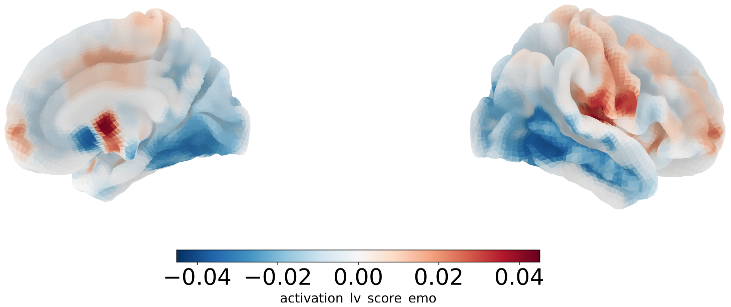

Supplementary Figure 7: Correlations between the Hurst-Psychopathology pattern and functional activation defined from a contrast between emotional and neutral faces. functional activation were not significantly correlated to the HPP after multiple comparison correction.

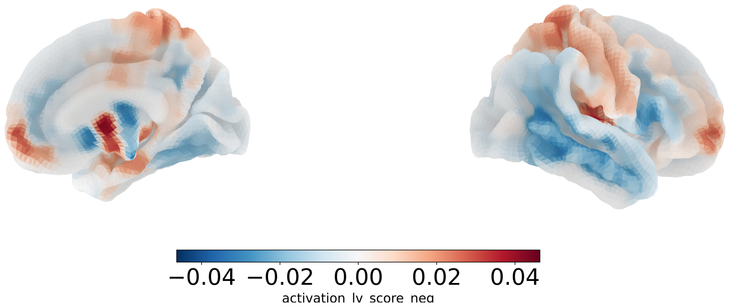

Supplementary Figure 8: Correlations between the Hurst-Psychopathology pattern and functional activation defined from a contrast between negative and neutral faces. functional activation were not significantly correlated to the HPP after multiple comparison correction.

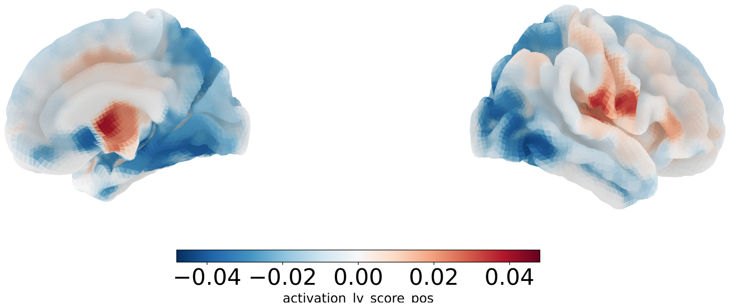

Supplementary Figure 9: Correlations between the Hurst-Psychopathology pattern and functional activation defined from a contrast between positive and neutral faces. functional activation were not significantly correlated to the HPP after multiple comparison correction.

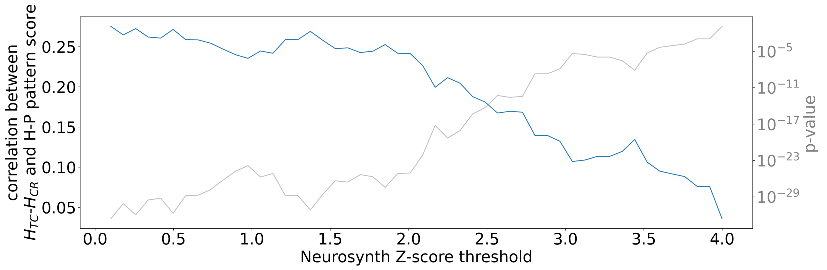

Supplementary Figure 10: Psychopathology is associated with relatively higher Hurst exponents in Task-Cognition areas relative to Cue-Response areas. The difference between the mean Hurst exponent for Task-Cognition (H_{TC}) and Cue-Response (H_{CR}) was correlated with participants' pattern scores (i.e., the degree to which each subject exemplifies the HPP during the EN-back task). This resulted in a significant positive correlation across all choices of Z-score threshold.

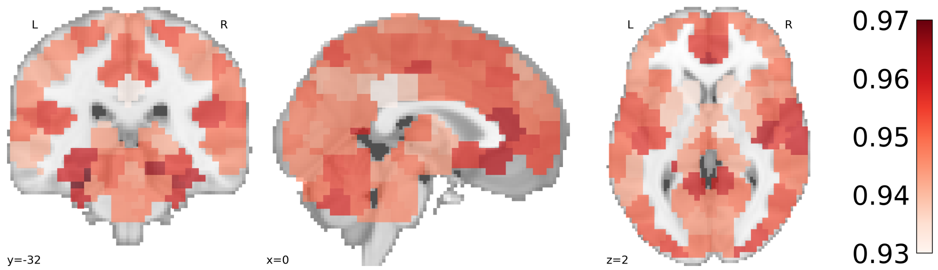

Supplementary Figure 11: Hurst R^2, mean for all participants. High R^2 values on average demonstrate good fits in the detrended fluctuation analysis, and suggest that the fMRI time series in these data are well described by a mono-fractal process, as opposed to a multi-fractal process.

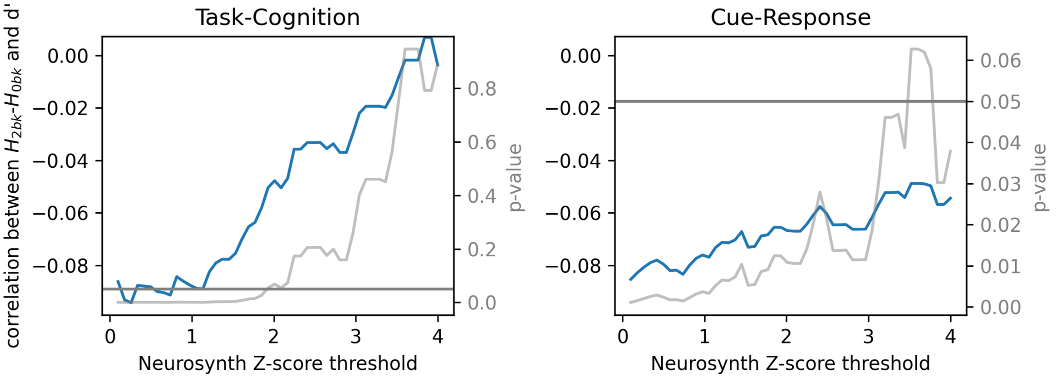

Supplementary Figure 12: Correlations between block level 2- vs. 0-back H contrast and d'. Correlations (blue) were computed across all choices of Z-score threshold used to define the task-cognition and cue-response ROIs from Neurosynth meta-analysis probabilistic activation maps. Significance of the correlations were assessed at all choices of threshold (gray). The p=0.05 level is shown as the gray horizontal line. We found evidence associating suppression of H with d' in both task-cognition and cue-response areas.

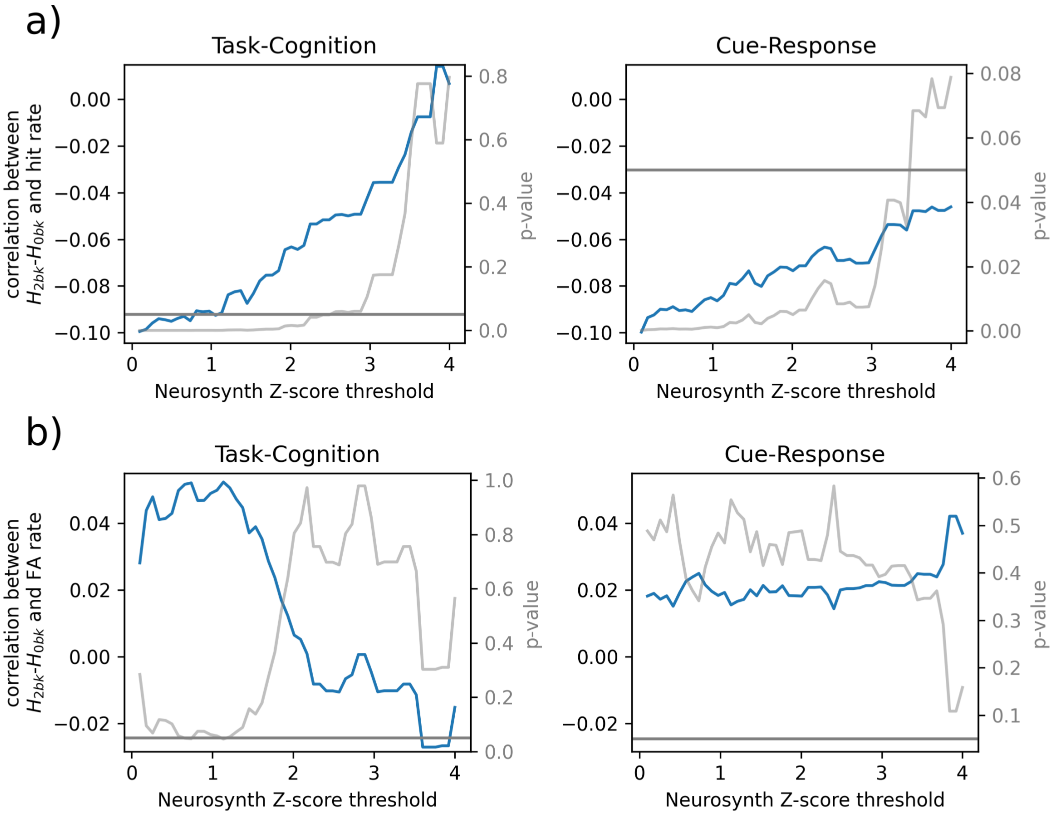

Supplementary Figure 13: Correlations between block level 2- vs. 0-back H contrast and hit rate (a, top) and false alarm rate, FA (b, bottom). Correlations (blue) were computed across all choices of Z-score threshold used to define the task-cognition and cue-response ROIs from Neurosynth meta-analysis probabilistic activation maps. Significance of the correlations were assessed at all choices of threshold (gray). The p=0.05 level is shown as the gray horizontal line. a/top) We found evidence associating suppression of H with hit rate in both task-cognition and cue-response areas. b/bottom) We found no evidence of a relationship between the 2- vs. 0-back H contrast and FA rate in neither task-cognition nor cue-response areas.

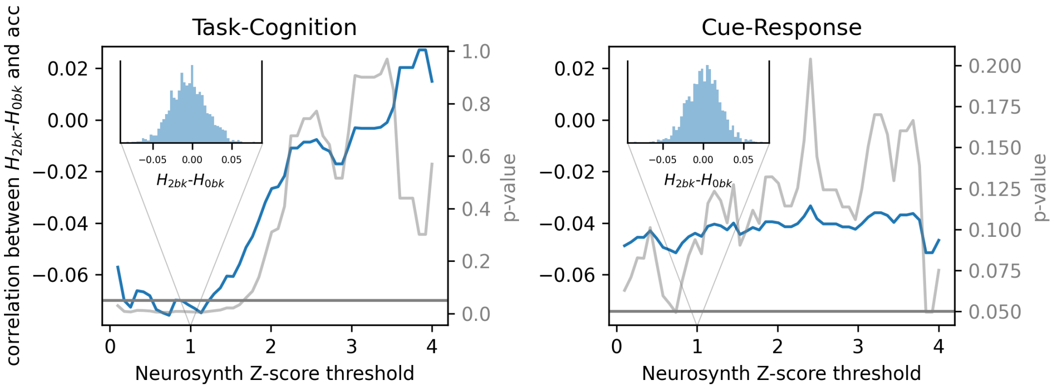

Supplementary Figure 14: Correlations between block level 2- vs. 0-back H contrast and 2-back accuracy (acc). Correlations (blue) were computed across all choices of Z-score threshold used to define the task-cognition and cue-response ROIs from Neurosynth meta-analysis probabilistic activation maps. Significance of the correlations were assessed at all choices of threshold (gray). The p=0.05 level is shown as the gray horizontal line. We found evidence associating suppression of H with acc in only task-cognition areas. Insets show the distribution of the 2-back vs. 0-back contrast for a Z-score threshold of 1.

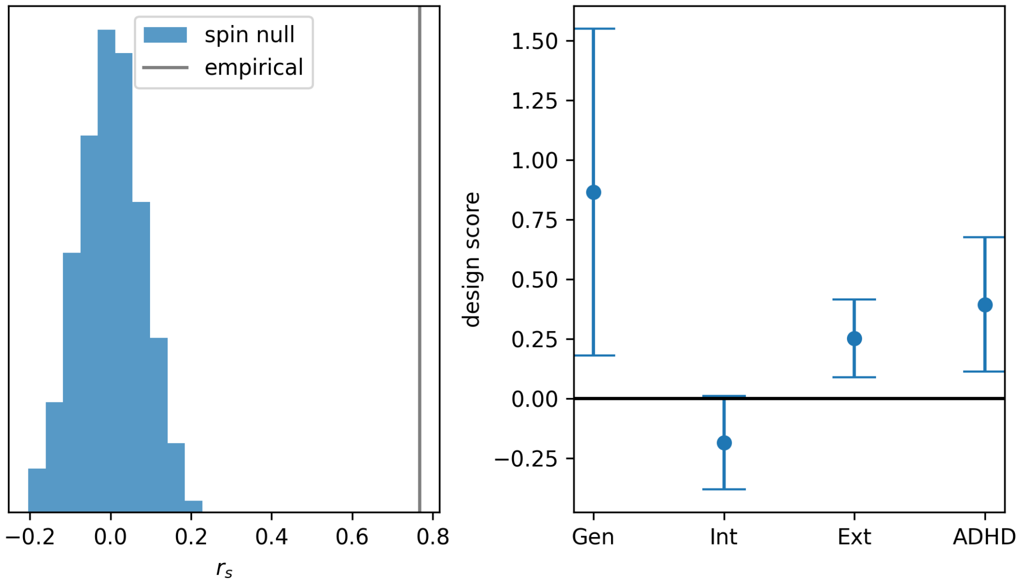

Supplementary Figure 15: Task structure does not drive the patterns of H. We re-ran the PLS analysis with H calculated from BOLD time series with the task block structure regressed out. Left) Brain loadings, i.e., the HPP is highly similar when task structure is and is not regressed out. The correlation is significant relative to spin nulls that account for spatial auto-correlation. Right) The design scores are highly similar whether or not task structure is accounted for.

Supplementary Tables

Supplementary Table 1: Demographics of included and excluded participants. Tests in bold are significant after false discovery rate correction (adopting a 5% false discovery rate) for six tests.

	Included Participants			Excluded Participants			Differences		
	Mean	SE	N	Mean	SE	N	t	d.f.	p <
Age in months	120.3	0.18	1839	118.7	0.07	10039	**8.5**		**2.075e-17**
Household size	4.9	0.05	1818	4.7	0.02	9781	**3.77**		**0.0002**
	n	%	N	n	%	N	χ^2	d.f.	p <
Sex (% female)	1018	0.55	1839	4664	0.46	10039	**48.95**	**1**	**2.6e-12**
Race-ethnicity			1839			10037	**146.74**	**4**	**1e-30**
Non - Hispanic white	1176	0.64		5006	0.5				
African - American	148	0.08		1636	0.16				
Hispanic	311	0.17		2100	0.21				
Asian	39	0.02		213	0.02				
Other	165	0.09		1082	0.11				
Income			1839			10039	**105.04**	**3**	**1.2e-22**
<50K	347	0.19		2877	0.29				
>50K & <100k	834	0.45		3731	0.37				
>100k	541	0.29		2530	0.25				
Not reported	117	0.06		901	0.09				
Maternal Education			1839			10039	**97.81**	**4**	**2.9e-20**
<HS Diploma	58	0.03		728	0.07				
HS Diploma/GED	885	0.48		3986	0.4				
Some College	139	0.08		1121	0.11				
College	503	0.27		2508	0.25				
Advanced Degree	139	0.08		1121	0.11				
Not reported	503	0.27		2508	0.25				

Supplementary Table 2: Demographics of included and excluded participants for HCP. Tests in bold are significant after false discovery rate correction (adopting a 5% false discovery rate) for four tests.

	Included Participants			Excluded Participants			Differences		
	Mean	SE	N	Mean	SE	N	t		p <
Age in years	28.6	0.14	703	29.2	0.16	503	**-2.68**		**0.0075**
	n	%	N	n	%	N	χ^2	d.f.	p <
Race-ethnicity			703			503	**44.96**	**5**	**1.5e-08**
Am.Indian/ Alaskan Nat.	1	0.0		1	0.0				
Asian/Nat. Hawaiian/Othr Pacific Is.	55	0.08		14	0.03				
Black or African-Am.	77	0.11		116	0.23				
More than one	18	0.03		14	0.03				
Unknown or Not Reported	10	0.01		13	0.03				
White	542	0.77		345	0.69				
Education (years)			702			502	**42.15**	**6**	**1.72e-07**
11.0	15	0.02		30	0.06				
12.0	81	0.12		95	0.19				
13.0	35	0.05		45	0.09				
14.0	88	0.13		66	0.13				
15.0	52	0.07		23	0.05				
16.0	312	0.44		178	0.35				
17.0	119	0.17		65	0.13				
Income			699			500	13.1	7	0.070
<10,000	44	0.06		46	0.09				
10K-19,999	52	0.07		51	0.1				
20K-29,999	90	0.13		63	0.13				
30K-39,999	79	0.11		58	0.12				
40K-49,999	65	0.09		61	0.12				
50K-74,999	153	0.22		97	0.19				
75K-99,999	96	0.14		58	0.12				
>=100,000	120	0.17		66	0.13				

Supplementary Table 3: Linear model predicting out-of-scanner performance from in-scanner performance and H in the HPP network.

**Dep. Variable:**	List Sorting Performance	**R-squared:**	0.101
**Model:**	OLS	**Adj. R-squared:**	0.100
**Method:**	Least Squares	**F-statistic:**	99.37
**Date:**	Sun, 22 Aug 2021	**Prob (F-statistic):**	1.27e-41
**Time:**	22:08:54	**Log-Likelihood:**	-6620.2
**No. Observations:**	1769	**AIC:**	1.325e+04
**Df Residuals:**	1766	**BIC:**	1.326e+04
**Df Model:**	2		

	**coef**	**std err**	**t**	**P> |t|**	**[0.025**	**0.975]**
**const**	79.5768	3.200	24.866	0.000	73.300	85.854
H **in HPP**	19.6572	4.317	4.553	0.000	11.190	28.125
**d' (in scanner)**	3.1741	0.260	12.227	0.000	2.665	3.683

**Omnibus:**	65.434	**Durbin-Watson:**	2.015
**Prob(Omnibus):**	0.000	**Jarque-Bera (JB):**	73.182
**Skew:**	-0.458	**Prob(JB):**	1.28e-16
**Kurtosis:**	3.392	**Cond. No.**	48.3

Supplementary Table 4: Demographics of study participants who had available 2-year follow up behavioral data for the in-scanner EN-back task. Tests in bold are significant after false discovery rate correction (adopting a 5% false discovery rate) for ten tests.

	Included Participants			Excluded Participants			Differences		
	Mean	SE	N	Mean	SE	N	t		p <
Age in months	120.2	0.25	888	120.5	0.25	951	-0.76		0.4459
Household size	4.9	0.1	880	4.8	0.05	938	1.33		0.1850
general factor	-0.0	0.03	888	-0.1	0.03	951	1.35		0.1773
Internalizing factor	0.1	0.02	888	0.1	0.02	951	0.9		0.3697
Externalizing factor	-0.1	0.02	888	-0.1	0.02	951	0.05		0.9585
ADHD factor	-0.1	0.02	888	-0.1	0.02	951	1.69		0.0917
	n	%	N	n	%	N	χ^2	d.f.	p <
Sex (% female)	457	0.51	888	561	0.59	951	**10.22**	**1**	**0.0014**
Race-ethnicity			888			951	11.39	4	0.0225
Non - Hispanic white	592	0.67		584	0.61				
African - American	61	0.07		87	0.09				
Hispanic	150	0.17		161	0.17				
Asian	21	0.02		18	0.02				
Other	64	0.07		101	0.11				
Income			888			951	9.54	3	0.0229
<50K	179	0.2		168	0.18				
>50K & <100k	371	0.42		463	0.49				
>100k	282	0.32		259	0.27				
Not reported	56	0.06		61	0.06				
Maternal Education			888			951	10.91	4	0.0276
<HS Diploma	24	0.03		34	0.04				
Hs Diploma/GED	438	0.49		447	0.47				
Some College	67	0.08		72	0.08				
College	219	0.25		284	0.3				
Advanced Degree	67	0.08		72	0.08				
Not reported	219	0.25		284	0.3				

Supplementary Table 5: Linear model predicting future in-scanner performance from baseline in-scanner performance, out-of-scanner performance and H in the HPP network.

**Dep. Variable:**	future d’	**R-squared:**	0.243
**Model:**	OLS	**Adj. R-squared:**	0.240
**Method:**	Least Squares	**F-statistic:**	90.37
**Date:**	Sun, 22 Aug 2021	**Prob (F-statistic):**	1.01e-50
**Time:**	22:08:31	**Log-Likelihood:**	-1163.5
**No. Observations:**	849	**AIC:**	2335.
**Df Residuals:**	845	**BIC:**	2354.
**Df Model:**	3		

	**coef**	**std err**	**t**	**P> |t|**	**[0.025**	**0.975]**
**const**	-1.6641	0.502	-3.317	0.001	-2.649	-0.679
H **in HPP (baseline)**	1.7510	0.584	2.998	0.003	0.605	2.897
**d’ (baseline)**	0.4162	0.036	11.537	0.000	0.345	0.487
**List sorting performance (baseline)**	0.0207	0.003	6.258	0.000	0.014	0.027

**Omnibus:**	284.338	**Durbin-Watson:**	1.984
**Prob(Omnibus):**	0.000	**Jarque-Bera (JB):**	3034.181
**Skew:**	-1.201	**Prob(JB):**	0.00
**Kurtosis:**	11.944	**Cond. No.**	2.23e+03

Supplementary Table 6: Mediation with Hurst as the mediator. h is the Hurst exponent in the HPP network. acc is 2-back accuracy. bf1-4 are extracted factor scores for, respectively, the general factor of psychopathology, the externalizing factor, the internalizing factor, and the ADHD factor.

lhs	op	rhs	est.std	se	z	pvalue	ci.lower	ci.upper
acc	~	bf1	−.056	.024	−2.323	.020	−.103	−.009
acc	~	bf2	.041	.024	1.696	.090	−.006	.089
acc	~	bf3	−.019	.024	−.774	.439	−.066	.029
acc	~	bf4	−.051	.023	−2.181	.029	−.097	−.005
acc	~	h	.223	.023	9.726	0	.178	.268
h	~	bf1	−.108	.024	−4.458	.00001	−.155	−.060
h	~	bf2	−.010	.025	−.386	.699	−.058	.039
h	~	bf3	−.026	.025	−1.060	.289	−.075	.022
h	~	bf4	−.094	.024	−3.946	.0001	−.140	−.047

Supplementary Table 7: Mediation with extracted bifactor scores as the mediator.h is the Hurst exponent in the HPP network. acc is 2-back accuracy. bf1-4 are extracted factor scores for, respectively, the general factor of psychopathology, the externalizing factor, the internalizing factor, and the ADHD factor.

lhs	op	rhs	est.std	se	z	pvalue	ci.lower	ci.upper
acc	~	bf1	−.056	.023	−2.385	.017	−.101	−.010
acc	~	bf2	.041	.023	1.782	.075	−.004	.087
acc	~	bf3	−.019	.023	−.809	.419	−.064	.027
acc	~	bf4	−.051	.023	−2.198	.028	−.097	−.006
acc	~	h	.223	.023	9.842	0	.178	.267
bf1	~	h	−.114	.024	−4.839	0.00000	−.160	−.068
bf2	~	h	−.010	.024	−.402	.688	−.057	.037
bf3	~	h	−.036	.024	−1.499	.134	−.083	.011
bf4	~	h	−.093	.024	−3.924	.0001	−.140	−.047

Supplementary Table 8: Harvard-Oxford labels, PLS loadings, bootstrap ratios and MNI coordinates for brain regions with absolute value bootstrap ratios > 2.5.

Harvard-Oxford Label	PLS loading	bootstrap ratio	MNI coordinates
Central Opercular Cortex	-0.1539	-4.65	(-58.0, -24.5, 18.5)
Superior Frontal Gyrus	-0.1278	-4.0	(24.7, 10.2, 57.5)
Inferior Frontal Gyrus, pars opercularis	-0.1209	-3.71	(-40.2, 8.1, 30.7)
Superior Frontal Gyrus	-0.1204	-3.93	(-21.9, 6.2, 63.3)
Middle Frontal Gyrus	-0.1159	-3.57	(28.5, -4.0, 52.8)
Precuneous Cortex	-0.1147	-3.54	(8.1, -66.5, 53.9)
Superior Frontal Gyrus	-0.1137	-3.52	(-16.3, -3.6, 68.0)
Superior Parietal Lobule	-0.1126	-3.47	(-16.7, -51.7, 66.0)
Middle Frontal Gyrus	-0.1114	-3.53	(-42.6, 22.5, 36.0)
Precentral Gyrus	-0.1091	-3.41	(-46.4, -0.5, 43.5)
Frontal Orbital Cortex	-0.1083	-3.31	(-27.2, 33.7, -15.9)
Superior Frontal Gyrus	-0.1083	-3.33	(20.7, -3.2, 66.3)
Central Opercular Cortex	-0.107	-3.22	(-41.6, -2.4, 8.1)
Cingulate Gyrus, anterior division	-0.1059	-3.24	(-7.8, -17.6, 43.7)
Postcentral Gyrus	-0.1042	-3.33	(62.9, -15.6, 17.9)
Postcentral Gyrus	-0.102	-3.14	(-27.0, -39.2, 63.9)
Central Opercular Cortex	-0.1017	-3.08	(41.6, -9.1, 13.9)
Cingulate Gyrus, posterior division	-0.0997	-3.14	(10.5, -27.3, 42.9)
Insular Cortex	-0.098	-3.07	(-35.7, -17.2, 1.1)
Lateral Occipital Cortex, superior division	-0.0968	-2.93	(-10.3, -65.8, 56.0)
Background	-0.0955	-3.03	(14.0, -56.1, -21.9)
Temporal Occipital Fusiform Cortex	-0.0948	-3.02	(37.3, -55.5, -17.2)
Frontal Pole	-0.0944	-2.98	(25.9, 59.6, -4.6)
Left Thalamus	-0.0942	-2.94	(-13.1, -23.1, 11.4)
Precentral Gyrus	-0.0923	-2.8	(-28.1, -6.4, 52.7)
Paracingulate Gyrus	-0.0913	-2.76	(-6.3, -0.7, 44.9)
Left Caudate	-0.0911	-2.77	(-13.2, 2.6, 16.3)
Lingual Gyrus	-0.0903	-2.84	(-24.3, -53.8, -9.2)
Precuneous Cortex	-0.0903	-2.87	(6.5, -52.8, 49.3)
Superior Parietal Lobule	-0.0894	-2.82	(26.5, -45.6, 64.5)
Postcentral Gyrus	-0.0893	-2.75	(-42.4, -18.4, 44.2)
Middle Frontal Gyrus	-0.0892	-2.85	(37.7, 3.0, 57.0)
Occipital Pole	-0.0888	-2.75	(-25.4, -95.0, 6.1)
Right Putamen	-0.0885	-2.7	(23.3, 9.0, 0.6)
Insular Cortex	-0.0885	-2.79	(33.1, 16.3, -8.0)

Cingulate Gyrus, posterior division	-0.0885	-2.77	(-5.4, -50.2, 19.6)
Background	-0.0872	-2.76	(18.6, -68.9, -27.2)
Frontal Pole	-0.0869	-2.72	(-45.8, 39.9, 4.1)
Occipital Pole	-0.0868	-2.74	(-8.5, -95.1, 0.5)
Left Putamen	-0.0859	-2.54	(-23.2, 7.9, 0.4)
Brain-Stem	-0.0847	-2.6	(-4.7, -25.1, -21.5)
Lateral Occipital Cortex, superior division	-0.0837	-2.69	(-27.3, -59.5, 56.7)
Background	-0.0832	-2.58	(29.9, -56.4, -27.9)
Superior Parietal Lobule	-0.0831	-2.63	(38.7, -46.0, 56.7)
Superior Parietal Lobule	-0.0829	-2.64	(40.5, -33.9, 44.7)
Juxtapositional Lobule Cortex	-0.0817	-2.6	(7.8, -13.6, 45.3)
Supramarginal Gyrus, anterior division	-0.0814	-2.65	(61.4, -22.8, 29.0)
Middle Frontal Gyrus	-0.0806	-2.56	(-36.2, 4.1, 56.0)
Frontal Pole	-0.0793	-2.56	(-25.8, 48.3, -14.0)
